# Closed-Loop Acoustic Stimulation of Slow Oscillations Using a Wireless EEG Headband: A Polysomnography-Validated Feasibility Study

**DOI:** 10.3390/s26206528

**Published:** 2026-10-15

**Authors:** Stefan L. Jongejan, Anna C. van der Heijden, Lucia M. Talamini

**Affiliations:** 1 Brain and Cognition, Department of Psychology, University of Amsterdam, Nieuwe Achtergracht 129-B, 1018WS Amsterdam, The Netherlandsl.m.talamini@uva.nl (L.M.T.); 2 Deep Sleep Technologies, Herengracht 584, 1017CJ Amsterdam, The Netherlands; 3 Amsterdam Brain and Cognition, University of Amsterdam, Nieuwe Achtergracht 129-B, 1018WS Amsterdam, The Netherlands

**Keywords:** modeling-based closed-loop neurostimulation, phase targeting, electroencephalography, EEG headband, EEG wearables, slow oscillations, slow-wave sleep, acoustic stimulation

## Abstract

Acoustic stimulation precisely timed during sleep can enhance slow oscillations (SOs), a hallmark of deep sleep that is critical for restorative sleep. To date, this approach has largely been restricted to laboratory settings using high-density electroencephalography (EEG). Wearable EEG systems featuring closed-loop acoustic stimulation could enable scalable, home-based approaches to modulating slow-wave sleep. Here, we tested whether a real-time modeling approach (M-CLNS) could be implemented using a wireless EEG headband with forehead electrodes in healthy participants. Acoustic stimulation was delivered during a single night of sleep in alternating stimulation and sham conditions. We evaluated the accuracy of SO phase targeting utilizing the EEG headband and assessed whether it could be used to detect stimulation-induced changes in oscillatory dynamics. The findings were validated against simultaneous polysomnographic (PSG) recordings as an independent reference. We found that SO phase was targeted consistently near the intended phase using the headband, despite reduced amplitude and signal quality compared with simultaneous PSG. Acoustic stimulation produced an immediate enhancement of SO dynamics, as well as sustained increases in SO density and power spectral density within the SO and delta frequency range (0.75–5 Hz) during deep sleep. These effects were apparent both in the EEG headband and PSG signals. These results demonstrate the feasibility of closed-loop acoustic stimulation using a wireless EEG headband, providing a foundation for future home-based and clinical applications.

## 1. Introduction

Deep sleep, also termed slow-wave sleep (SWS), is a fundamental component of healthy sleep, supporting essential growth and recovery processes that sustain physical and mental health. SWS has been associated with immune function [1,2], the build-up of energy molecules [3], cardiovascular health [4], glucose homeostasis [5], and brain waste clearance [6]. Reductions in SWS have been described in several conditions, including sleep disorders like insomnia [7,8] and sleep apnea [9], psychiatric conditions such as post-traumatic stress disorder [10] and depression [11], neurodegenerative diseases like Alzheimer’s [12] and Parkinson’s disease [13], and somatic syndromes including chronic fatigue syndrome [8]. SWS also declines naturally across the lifespan [14], making it particularly relevant in the context of the widespread prevalence of poor sleep in modern societies [15,16,17,18,19,20]. Approaches that can selectively enhance SWS may therefore have broad relevance for supporting sleep quality and maintaining health across the general population.

Despite the high prevalence of SWS deficits, commonly prescribed treatments do not reliably enhance deep sleep. Standard hypnotics, including benzodiazepines, primarily increase NREM stage 2 sleep while potentially reducing deep sleep [21]. Consistent with this shift in sleep architecture, chronic use has been associated with reduced delta power [22]. These pharmacological interventions also have important side effects [23], including habituation, dependence [24,25], and impaired cognitive performance [26]. Cognitive behavioral therapy for insomnia provides a non-pharmacological alternative for improving sleep quality [27], but it is costly, time-consuming, and only moderately effective. Moreover, it does not directly target a lack of SWS. These limitations motivate the development of accessible interventions that specifically modulate the neurophysiological activity underlying deep sleep.

A promising approach to enhancing SWS is the non-invasive delivery of acoustic stimulation aligned with slow oscillations (SOs; approximately 0.5–1.5 Hz), an electrophysiological hallmark of SWS [28,29]. Previous studies have shown that acoustic stimulation can enhance SO activity during the seconds immediately following stimulation or during rhythmic stimulation at a frequency within the SO range, typically approximately 1 Hz [29,30,31,32,33,34,35,36]. However, the effects of rhythmic stimulation appear to be self-limiting, diminishing with successive stimuli [37]. One possible explanation is that stimuli not targeted to the appropriate phase interfere with intrinsic oscillatory brain dynamics. Consistent with this interpretation, non-phase-targeted acoustic stimuli have been shown to disrupt traveling slow waves [38]. The effectiveness of acoustic stimulation may therefore depend on accurately determining the ongoing EEG dynamics and delivering each stimulus at the appropriate oscillatory phase.

Modeling-based closed-loop neurostimulation (M-CLNS) was developed to meet this requirement. M-CLNS models and predicts raw neurophysiological signals with millisecond temporal resolution, enabling stimuli to be targeted to selected phases of a wide range of brain oscillations [39]. The method does not require participant-specific training or habituation nights. M-CLNS has been used to precisely phase-lock individual acoustic stimuli to the onset of SOs, producing both immediate stimulus-locked increases in SO activity and longer-term effects consistent with overall sleep deepening [40,41,42].

To date, acoustic sleep stimulation using M-CLNS has predominantly been investigated in laboratory settings using full polysomnography (PSG) and laboratory-grade EEG equipment [40,41,42]. Although these systems provide high-quality multichannel recordings, they are laborious, requiring trained personnel, wired acquisition hardware, and dedicated sleep-laboratory facilities. Their cost and complexity restrict repeated measurements, studies involving large numbers of participants, and prolonged use in clinical or home environments. Broader assessment of M-CLNS therefore requires a more compact and user-friendly sensing system that retains the real-time signal access and temporal performance needed for closed-loop stimulation.

Miniaturized wireless EEG headbands could provide such a sensing system, enabling M-CLNS to incorporate EEG acquired through a more compact and accessible wearable platform. This could facilitate repeated measurements and extend closed-loop stimulation beyond the sleep laboratory, into home situations and other relevant environments [43]. However, applying M-CLNS over wearable EEG also presents important challenges. Wearable devices generally use fewer electrodes, more restricted electrode locations, and wireless-transmission hardware. Their EEG signals may consequently differ from PSG recordings because of electrode placement and referencing. Moreover, the reduced size, cost, as well as constructional compromises to allow self-applicability by lay people tend to come at the cost of increased motion and sweat artifacts, hardware filtering, and transmission delays. For closed-loop stimulation, validation must therefore extend beyond general EEG signal quality. The wearable system must provide access to the raw signal with sufficient reliability and temporal resolution, sufficiently support accurate real-time prediction and stimulus timing, and capture the neurophysiological responses produced by stimulation.

Among these hardware characteristics, electrode configuration is particularly relevant for wearable sleep EEG. Electrode placement must balance signal quality with comfort, ease of use, and suitability for prolonged recordings. Forehead placement is particularly attractive because it avoids hair-covered scalp regions, facilitates self-application, and provides access to frontal EEG, where slow oscillations are particularly prominent [44]. Solid-gel electrodes on the hair-free forehead may also provide stable contact during overnight recordings, while their visibility to the wearer can support correct sensor placement. These practical and physiological considerations motivate the use of a forehead-based EEG configuration for evaluating the wearable implementation of M-CLNS.

In this study, we investigate whether M-CLNS with acoustic stimulation of SOs can be implemented using EEG acquired from a wireless EEG headband with forehead electrode derivations. We use the research-grade ZMax device (Hypnodyne Corp., Sofia, Bulgaria) [45,46], which provides real-time access to the raw EEG signal and has previously been validated against PSG for signal quality [45]. The headband-derived EEG is used for real-time modeling and prediction of SO dynamics. Simultaneous PSG is recorded during a single laboratory night in healthy participants to serve as an independent reference. The primary aims are twofold: to determine whether forehead-derived EEG from the wireless headband can support accurate, real-time targeting of acoustic stimuli to the SO phase, and whether the resulting stimulation-related brain response can be detected in the headband EEG.

## 2. Materials and Methods

### 2.1. Participants

Data were recorded from 26 healthy participants (14 males, mean ± SD age 21.23 ± 2.76 years), of whom 16 participants (8 males, 21.13 ± 2.22 years) could be retained for analyses after exclusions based on headband signal quality (*n* = 5) and procedural or sleep-related criteria (*n* = 5; Table 1). None of the participants reported diagnosed sleep, neurological or psychiatric disorders or the use of prescription drugs known to affect sleep. Participants were asked to abstain from alcohol consumption and recreational drug use for 24 h and to avoid caffeine consumption for at least 7 h preceding the experiment. Moreover, participants were asked to go to bed no later than 12:00 a.m. on the night prior to the experiment, and to awaken no later than 08:00 a.m. the next morning. All participants provided written informed consent. This study was approved by the Ethics Committee of the University of Amsterdam.

### 2.2. EEG Headband Data Acquisition

EEG was acquired from the wireless ZMax EEG headband (Hypnodyne Corp., Sofia, Bulgaria), positioned just above the eyebrows on the flattest available surface of the forehead. The ZMax headband was selected for this study as it is one of the few commercially available EEG wearables with low-latency access to real-time data, while remaining wireless [46]. Left and right forehead EEG derivations, approximating F7-Fpz and F8-Fpz, were captured using Ag/AgCl solid hydrogel electrodes (Hypnodyne Corp., Sofia, Bulgaria). In addition to EEG, 3-axis acceleration and photoplethysmography were recorded by sensors in the headband; those signals were not used for closed-loop control or for the offline analyses reported here.

Data from all headband sensors were wirelessly transmitted to a receiver dongle, connected to a USB hub located less than 1 m behind the participant’s pillow. The signals were sampled at 256 Hz with a bandwidth of 0.1–128 Hz and acquired through a Transmission Control Protocol socket. Data recording and stimulus decisions were conducted on a dedicated computer using a custom version of EventIDE (version 21.11.2019; Okazolab Ltd., Delft, The Netherlands), visual programming software for designing and executing experiments using real-time and low-latency signal processing.

The referencing scheme of the EEG headband results in a signal polarity inversion relative to standard mastoid-referenced EEG. Our algorithm targeted the onset of the positive deflection of the recorded headband signal, which corresponds to the onset of the negative deflection of the slow oscillation as defined by standard mastoid referencing.

### 2.3. PSG Data Acquisition

PSG data, consisting of 64-channel EEG, vertical- and horizontal-electrooculography, and chin electromyography were recorded using a Refa8 72-channel DC amplifier (TMSi, Oldenzaal, The Netherlands). EEG was captured using WaveGuard caps (ANT, Enschede, The Netherlands) with separate sintered AgCl reference electrodes (MedCat, Klazienaveen, The Netherlands) placed on the earlobes (A1, A2). Signals were sampled at 512 Hz and recorded on a dedicated computer using a customized version of TMSi Polybench (version 1.34.1.4456; TMSi, Oldenzaal, The Netherlands). EEG impedances were verified to be below 10 kΩ at the onset of recording.

The data acquired from both the headband and PSG were time-synchronized using markers created in EventIDE and sent to the PSG amplifier for simultaneous recording in the PSG data. Identifiable markers were recorded at the start and end of the sleep recording, as well as at the start and end of each experimental block and at the onset of each stimulus presentation.

### 2.4. M-CLNS Algorithm

The M-CLNS algorithm, developed at our lab in collaboration with Okazolab Ltd. (Delft, The Netherlands), was employed to continuously perform non-linear sine fitting on the most recent 1.6 s calculation windows of the headband’s raw F7-Fpz signal. Stimulus release was targeted halfway through the positive-going deflection, defined as 0° phase, within the forecasted signal in the SO frequency range of 0.5–1.5 Hz. Acoustic stimuli were only released when the best-fitting estimated model had a suprathreshold fit (max average fit error 0.2) and the targeted phase was predicted to occur 24–34 ms in the future (search window), based on the best-fitting estimated model [39]. This search window for the target phase compensates for loop lag in the entire hardware/software system [39].

### 2.5. Stimulation Protocol

Acoustic stimuli were presented using Creative GigaWorks T20 Series II stereo speakers (Creative Technology Ltd., Singapore) and consisted of faded-in 100 ms pink noise pulses, calibrated to be 42.5 dB(A) equivalent using a Rion NA-27 sound level meter (RION Co., Ltd., Kokubunji, Japan). The calibration point, at approximately 50 cm distance from the midpoint between the left and right speaker (located ~56 cm apart), was chosen to approximate participant head location during sleep.

PSG and headband signals were monitored in real time by the experimenters. The M-CLNS SO detection algorithm was manually enabled at SWS onset. During the ensuing three-hour interval, acoustic stimuli were presented phase-locked to SOs, while stimulus presentation was manually paused whenever light sleep, REM sleep, movement, or arousals were observed. Stimulus presentation occurred in 30 s blocks of either acoustic stimulation (STIM) or muted stimulation (SHAM). SHAM blocks used the same phase-locked targeting and trigger timing as STIM, but the acoustic stimulus was muted and therefore inaudible. STIM and SHAM blocks were alternated randomly and were interspersed with 30 s washout blocks. The onset of presented stimuli was recorded in EventIDE and sent to the PSG system for simultaneous recording in Polybench.

### 2.6. Procedure

Participants slept a single night at the Amsterdam Sleep and Memory Lab. They arrived at 09:00 p.m. and were asked to perform a quick hearing test to confirm the acoustic stimulation’s sound level was within their hearing range. Participants went to bed between 11:00 and 11:30 p.m. and were given the opportunity to sleep a minimum of 8 h, while simultaneously wearing both the EEG headband and PSG equipment. No information was provided on the acoustic stimulation that was to occur during sleep.

### 2.7. Data Analysis

#### 2.7.1. Pre-Processing

Offline data was pre-processed using EEGLAB 14.1.2b [47] in MATLAB R2016a. The PSG EEG data were down-sampled to 256 Hz and 0.1–49 Hz band-pass filtered, and bad signals were interpolated (2.32% ± 3.53% of signals) and re-referenced to the average of A1 and A2 for sleep stage scoring. Additionally, F7 was re-referenced to Fpz for comparison to the concomitant headband forehead derivation, to assess consistency and possible differences between the two signals. The same band-pass filter was applied to the headband EEG data.

Sleep recording quality was evaluated through visual inspection of the signals and spectrograms (0.5–48 Hz), which were created using the Chronux 2.11 Spectral Analysis toolbox [48] in MATLAB. Signal stretches containing artifacts were removed from both PSG and headband EEG before further analyses were performed.

Stimulus presentation blocks from F7-Fpz for both the headband and PSG data were collapsed into STIM and SHAM data sets for each participant. These data sets were then exported as EDF files using EEGLAB. The EDF files were further processed and analyzed in Python (3.11.9) using the Pandas (2.2.3), NumPy (2.1.3), MNE (1.9.0), SciPy (1.15.2), PyCircStat (0.0.2), Astropy (7.0.1), Statsmodels (0.14.4), Matplotlib (3.10.0), and YASA (0.6.4) libraries [49,50,51,52,53,54,55,56,57,58].

#### 2.7.2. Phase Accuracy

First, stimulus presentation accuracy for the STIM and SHAM conditions was calculated. For this purpose, the data sets, containing stimulus onset markers, were band-pass filtered (0.5–1.5 Hz FIR; MNE), and the analytic signal was obtained via Hilbert transform (SciPy). Instantaneous phase was extracted at each stimulus marker, and π/2 was subtracted to account for the phase shift associated with the Hilbert transform. Subsequently, the phase offset from the targeted phase was calculated in degrees for each corrected instantaneous phase. Circular statistics (mean, median, and standard deviation; Astropy and PyCircStat) were computed across STIM and SHAM for both the headband F7-Fpz and PSG F7-Fpz to assess accuracy (mean/median phase offsets) and precision (phase offset standard deviations) of stimulus presentations [39]. Mean vector length (MVL, a measure of phase concentration; PyCircStat) and Rayleigh tests (testing for deviation from a uniform phase distribution; PyCircStat) were additionally computed on the phase at each stimulus marker, separately for STIM and SHAM, in both the headband F7-Fpz and PSG F7-Fpz signals. Bootstrap 95% confidence intervals were obtained for the circular mean and for MVL (100,000 resamples). Pairwise differences in circular mean phase and in MVL, between STIM and SHAM and between the headband and PSG, were assessed with permutation tests (100,000 permutations), which require no parametric assumptions about the shape of the circular distribution. Permutation *p* values were adjusted across the four planned comparisons within each test with the Hochberg step-up procedure with a significance threshold of 0.05. The magnitude of MVL differences was expressed as the effect size (Δr), reported with a bootstrap 95% confidence interval (100,000 resamples).

#### 2.7.3. Event-Related Potentials

To assess stimulus-locked effects, event-related potentials (ERP; MNE) were analyzed, again for headband F7-Fpz and PSG F7-Fpz. All STIM and SHAM data sets were band-pass filtered (0.5–1.5 Hz FIR; MNE), and epochs were extracted from −1 to 2 s around the stimulus onset markers. A baseline correction was applied using the pre-stimulus interval (−1 to 0 s). ERPs were then averaged across trials within each participant and condition. Grand average ERPs across all participants were then calculated for both the STIM and SHAM conditions. Within each device (headband and PSG), STIM and SHAM were compared with a cluster-based permutation test using 100,000 within-subject sign-flip permutations across time points after stimulus onset with a significance threshold of 0.05. Following the same method, the STIM and SHAM conditions were compared across devices.

#### 2.7.4. Power Spectral Density

Longer-term effects of M-CLNS with acoustic stimulation on the sleep EEG were assessed through PSD and the detection of discrete slow oscillations and slow sleep spindles (see 2.7.5). PSD was estimated using Welch’s method (SciPy), with power calculated in 0.25 Hz bins across the frequency range of 0.25–35 Hz. These bins were chosen to provide a fine-grained resolution of power, allowing for the detection of subtle differences in oscillatory activity. Two participants exceeded the Tukey fence (Q3 + 1.5 × IQR) in both conditions and were excluded from the PSD analyses (*n* = 14). To compare power between the STIM and SHAM conditions, we conducted paired samples *t*-tests (SciPy) when the data met normality assumptions and Wilcoxon signed-rank tests (SciPy) when the data did not meet normality assumptions, for each frequency bin across all participants. To control multiple comparisons, we applied a false discovery rate correction (Benjamini–Hochberg; Statsmodels) with a significance threshold of 0.05. Additionally, we calculated the percentage difference in power between STIM and SHAM for each frequency bin to further illustrate power changes between conditions.

#### 2.7.5. Slow Oscillations and Spindle Characteristics

Longer-term effects of acoustic stimulation on slow oscillations and sleep spindles were assessed through automated detection of discrete slow oscillation and spindle events. Given the frontal placement of the headband and the occurrence of slow spindles in the frontal region [59], only slow spindles were assessed. Fast spindles, which are typically observed centroparietally [59], were not evaluated, as the headband did not contain centroparietal derivations.

Slow oscillations and slow spindles were analyzed for each participant and signal (PSG F7-Fpz and headband F7-Fpz) for concatenated STIM and SHAM blocks separately. Slow waves were detected using YASA with 40% reduced amplitude thresholds for both PSG F7-Fpz and headband EEG F7-Fpz [45], to account for the low amplitudes associated with the frontal referencing scheme. Average slow wave characteristics, including peak-to-peak amplitude, negative-peak amplitude, positive-peak amplitude, slope, and density, were determined per participant. Similarly, slow sleep spindles (9–12 Hz) were detected using YASA’s default settings, which do not include an amplitude threshold. The normality of the data was assessed through Shapiro–Wilk tests and visual inspection of Q-Q plots. To compare characteristics between the STIM and SHAM conditions, we conducted paired samples *t*-tests (SciPy) when the data met normality assumptions, and Wilcoxon signed-rank tests (SciPy) when the data did not meet normality assumptions, for each characteristic across all participants in whom slow waves or slow sleep spindles could be detected. To control multiple comparisons, we applied a false discovery rate correction (Benjamini–Hochberg; Statsmodels) with a significance threshold of 0.05.

#### 2.7.6. Sleep Composition

Sleep stages were manually scored based on the PSG recording. Sleep macro-architecture (total sleep time and the percentage of total sleep time in NREM1, NREM2, NREM3, and REM) was summarized from those stages.

To assess whether acoustic stimulation fragmented sleep, the PSG sleep stage in the epoch immediately after the last stimulus of each STIM or SHAM block was compared with the stage of the preceding epoch. Per-participant proportions of blocks with an unchanged stage, a transition to a lighter sleep-wake stage (i.e., N2, N1, or wake), or a transition to REM were compared between STIM and SHAM using paired samples *t*-tests (SciPy) when the data met normality assumptions and Wilcoxon signed-rank tests (SciPy) when the data did not meet normality assumptions (Shapiro–Wilk), with a false discovery rate correction (Benjamini–Hochberg; Statsmodels) with a significance threshold of 0.05.

## 3. Results

### 3.1. Headband EEG Signal Quality and Sleep Architecture

Of the 26 enrolled participants, 16 were retained for the final analyses, while 10 were excluded. Five participants were excluded due to headband signal quality. This entailed poor signal quality for more than 98% of the recording (*n* = 2) or substantial quality issues during stimulation (*n* = 3). Five additional participants were excluded based on procedural or sleep-related criteria, specifically, failure to complete the sleep interval (*n* = 1), absence of slow-wave sleep (*n* = 1), insufficient spontaneous slow-wave sleep continuity prior to stimulation (*n* = 2), or computer issues (*n* = 1). Visual inspection of headband and PSG F7-Fpz signals for the hallmark oscillations of NREM sleep, slow oscillations and sleep spindles, showed that slow-wave patterns were recognizable in both recordings. Relative to the simultaneous PSG, visual inspection indicated that the raw headband signal consistently exhibited lower amplitude and more frequent eye-movement, high-frequency power, and sweat artifacts.

The final analyses included 16 participants, who slept for an average of 482.69 ± 34.35 min (mean ± SD), with 22.68% ± 6.47% of sleep time spent in deep sleep (NREM3) (Table 2). Sleep stage remained stable following stimulation. PSG sleep stage was unchanged from the epoch before the last stimulus to the subsequent epoch in 95.64% of STIM blocks and 93.98% of SHAM blocks. Transitions to lighter sleep stages were infrequent and did not differ between conditions (STIM: 2.87%; SHAM: 4.19%; *p* = 0.600). Transitions to REM sleep likewise did not differ between conditions (*p* = 0.600).

### 3.2. Slow Oscillation Phase-Targeting Accuracy

**Figure 1:**
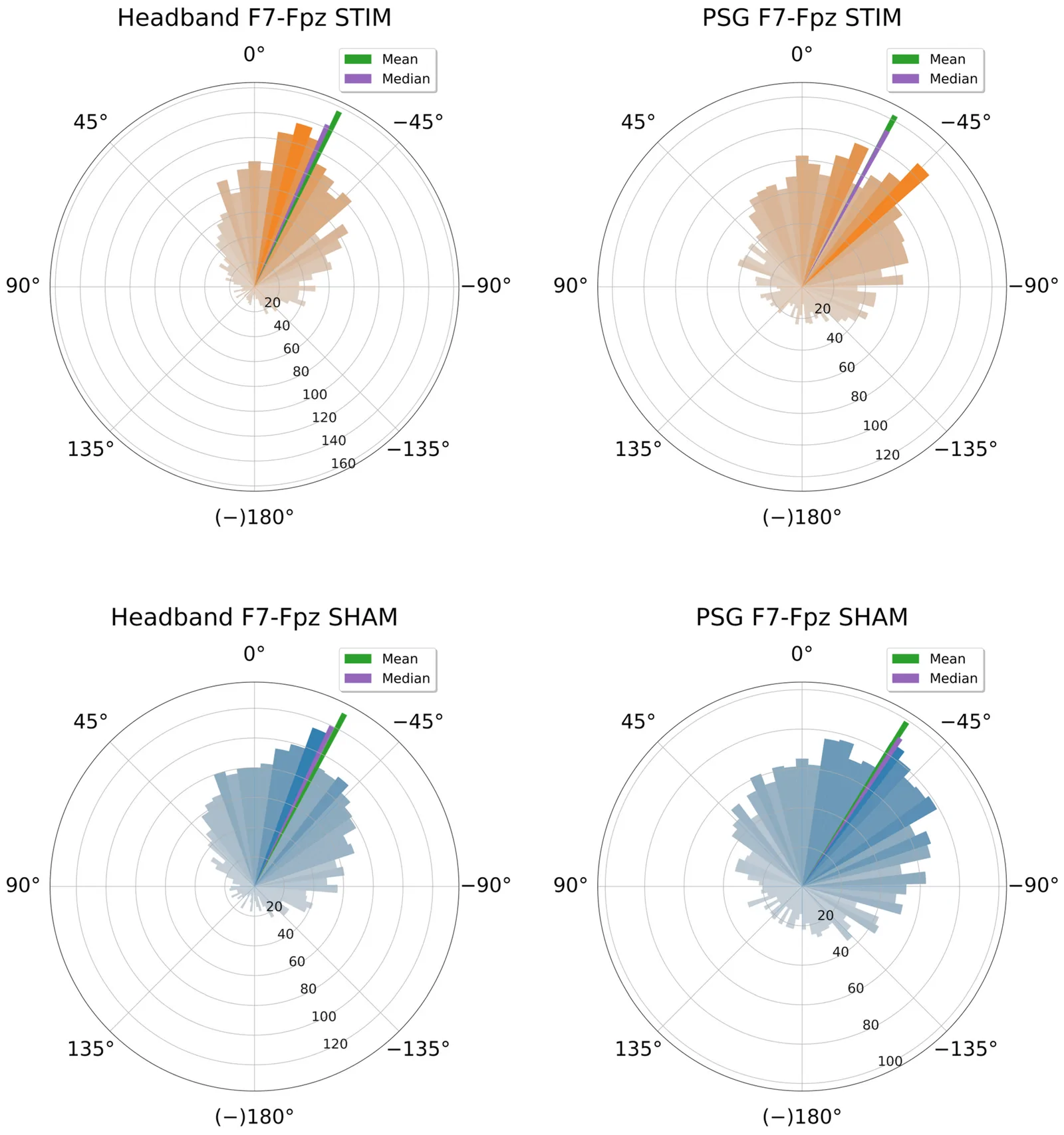
Phase targeting performance for slow oscillations recorded from a forehead derivation using an EEG headband. Circular histograms display the distribution of binned stimulus marker offsets (the difference between stimulus and target phase in degrees) for STIM (orange) and SHAM (blue) conditions, measured in both PSG F7-Fpz and EEG headband F7-Fpz. The mean and median stimulus marker offsets are indicated by the green and purple lines, respectively. Darker shades indicate a greater number of stimulus markers in a bin, and lighter shades indicate fewer markers.

A total of 2665 and 2558 stimuli were presented during the STIM and SHAM blocks, respectively, corresponding to an average of 6.07 and 5.99 stimuli per 30 s block. Circular means for phase targeting accuracy and precision were −25.83° (CI: −28.33° to −23.34°; SD 54.85°; median −24.26°) for headband STIM, −27.55° (CI: −30.42° to −24.67°; SD 57.17°; median −25.80°) for headband SHAM, −28.54° (CI: −32.32° to −24.77°; SD 63.68°; median −28.67°) for PSG STIM, and −32.47° (CI: −36.85° to −28.11°; SD 65.62°; median −33.52°) for PSG SHAM (Figure 1).

Rayleigh tests were significant (*p* < 0.0001) in all four conditions, indicating that stimulus phases were non-uniformly distributed and clustered around preferred phases of the slow oscillation. Mean vector length was 0.54 (CI: 0.52 to 0.56) for headband STIM, 0.50 (CI: 0.48 to 0.52) for headband SHAM, 0.38 (CI: 0.36 to 0.41) for PSG STIM, and 0.34 (CI: 0.32 to 0.37) for PSG SHAM, indicating moderate phase concentration.

Mean phase did not differ between STIM and SHAM for either the headband (difference: 1.72°, *p* = 1.000) or PSG (difference: 3.93°, *p* = 0.357). Likewise, mean phase did not differ between headband and PSG recordings during STIM (2.71°, *p* = 0.647) or SHAM (4.92°, *p* = 0.051), indicating similar stimulus timing across recording modalities and stimulation conditions.

Phase concentration was higher for the headband than for PSG during both STIM (Δr = 0.160, 95% CI 0.128–0.191, *p* < 0.00001) and SHAM (Δr = 0.158, 95% CI 0.125–0.191, *p* < 0.00001), consistent with the fact that stimulation timing was determined from the headband signal. Within the headband recordings, phase concentration was slightly higher during STIM than SHAM (Δr = 0.040, 95% CI 0.009–0.070, *p* = 0.035), whereas the corresponding PSG difference was not significant (Δr = 0.038, 95% CI 0.004–0.073, *p* = 0.119). The somewhat tighter phase concentration in the headband in the STIM versus SHAM condition might reflect a snowball effect, whereby stimulation induces more prominent slow oscillations that, in turn, enhance subsequent slow-oscillation targeting.

Overall, these findings indicate consistent phase targeting around the intended phase in both the headband and PSG recordings, with mean phase directions close to the target and comparable across devices and conditions.

### 3.3. Event-Related Potentials

**Figure 2:**
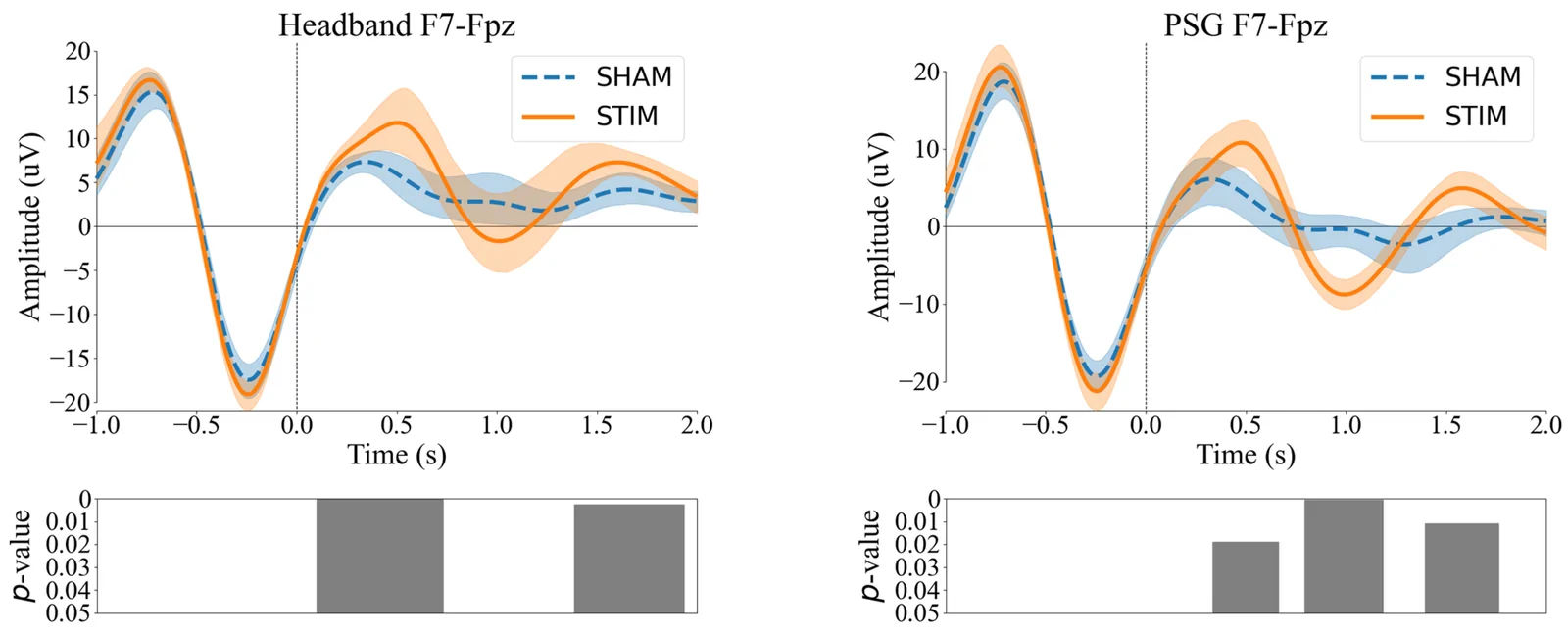
Stimulus-locked effects of M-CLNS with acoustic stimulation targeting SOs in forehead-derivation EEG from a headband and simultaneous PSG. Grand average ERP plots are presented comparing STIM and SHAM conditions for headband F7-Fpz and PSG F7-Fpz signals, with corresponding 95% confidence intervals. STIM versus SHAM differences were assessed with a cluster-based permutation test (100,000 within-subject sign-flip permutations). The significance threshold was *p* < 0.05.

Grand average ERPs reveal an enhancement of an SO-like dynamic in the STIM compared to the SHAM condition, in both the headband F7-Fpz and PSG F7-Fpz signals (Figure 2).

A cluster-based permutation test identified three clusters in the headband signal: a significant positive cluster at 98–730 ms, followed by a trend-level negative cluster at 852–1129 ms (*p* = 0.050), and a subsequent significant positive cluster at 1387–1934 ms. The PSG signal showed a similar pattern with three significant clusters. First, a positive cluster at 332–660 ms, next, a negative cluster at 793–1184 ms, and a second positive cluster at 1395–1762 ms. Cluster time ranges are reported in Table 3. Overlap of the corresponding headband and PSG clusters was 51.90%, 100.00%, and 67.09%, respectively (Table 3).

Together, these results highlight an enhancement of the SO-like deflection. This may indicate increased amplitude or occurrence of slow oscillations, or a more stereotyped morphology of the stimulation-evoked slow wave leading to stronger phase alignment.

### 3.4. Power Spectral Density

**Figure 3:**
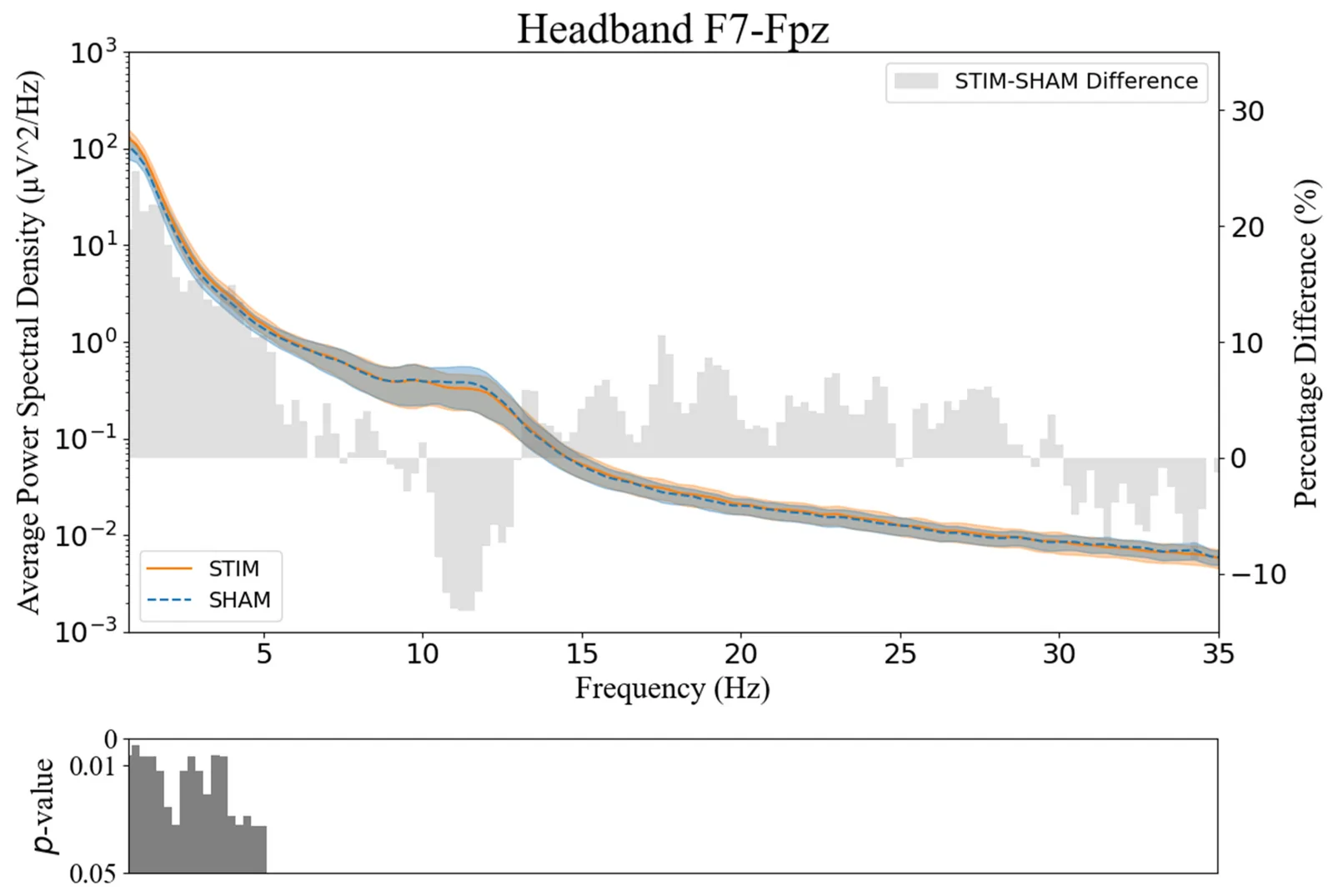

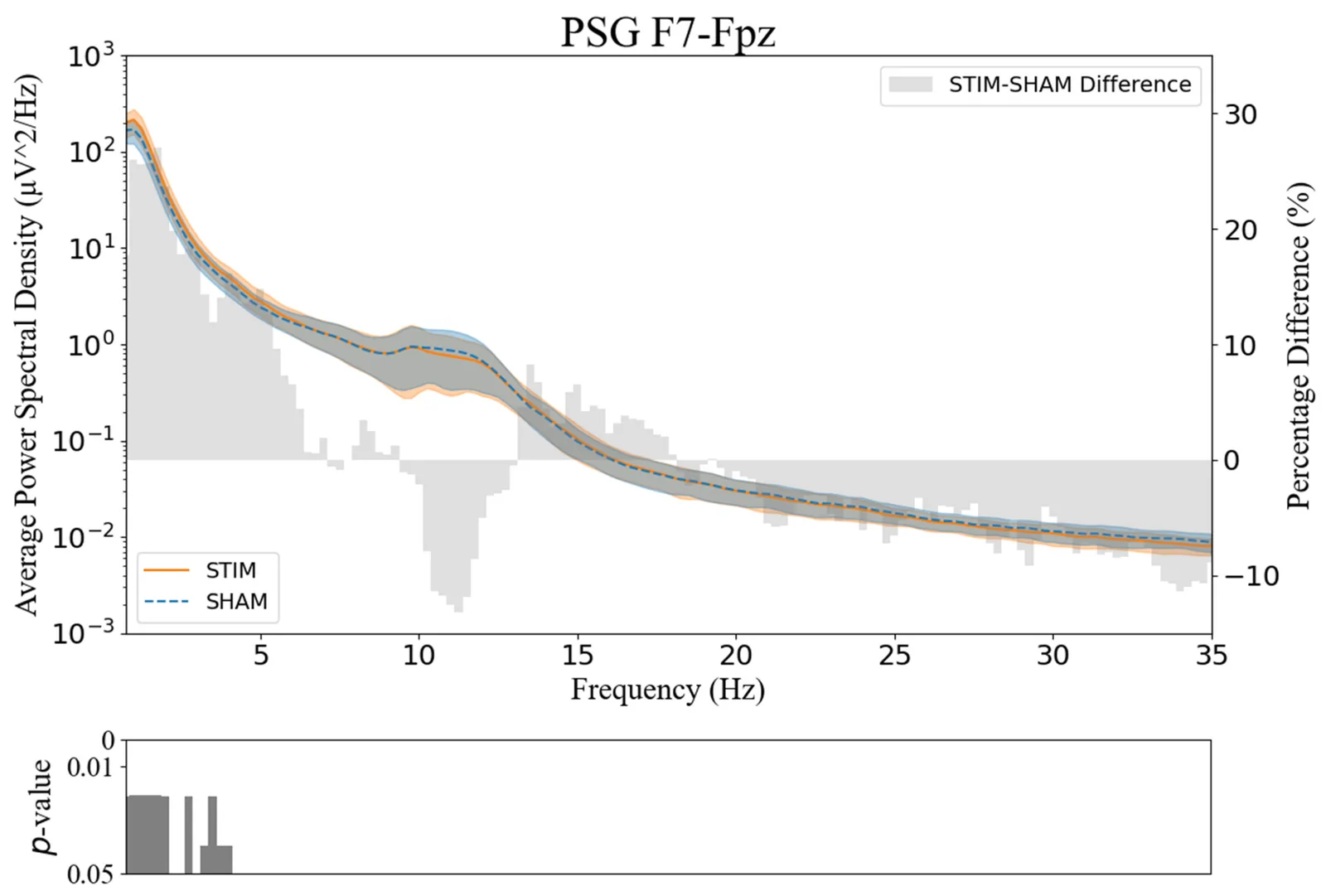
Power spectral density (PSD) plots comparing STIM and SHAM conditions in 0.25 Hz bins across the 0.25–35 Hz frequency range, for both headband F7-Fpz and PSG F7-Fpz signals. The plots include 95% confidence intervals and percentage differences in power between STIM and SHAM. Significant differences, based on FDR-corrected paired samples *t*-tests or Wilcoxon signed-rank tests, are indicated in accompanying bar plots.

To assess overall effects of stimulation on brain activity, we determined differences in PSD (frequency range between 0.25 and 35 Hz) between the STIM and SHAM condition. Headband EEG showed statistically significant power increases across the SO and delta range from 0.75 to 5 Hz in STIM relative to SHAM (Figure 3; Table 4). Similarly, the PSG F7-Fpz data showed statistically significant increases from 0.75 to 2 Hz, at 2.75 Hz, and from 3.25 to 4 Hz. All tested frequency bins are reported in Supplementary Table S1.

The average increase in power, based on the percentage difference between sham and stimulation across significantly increased frequency bins in the delta range (0.75–4 Hz) was 17.45% ± 3.74% for the headband and 20.03% ± 5.66% for PSG. Considering only the SO range (0.75–1.5 Hz), the average increase in bin power across frequency bins showing a significant increase was 21.88% ± 2.11% for the headband and 23.74% ± 4.04% for PSG (Table 4).

These findings show that acoustic stimulation with M-CLNS can enhance SO and delta activity across sleep, as apparent in both the headband and PSG recordings.

### 3.5. Slow Oscillation and Spindle Characteristics

A second overall analysis assessed effects of stimulation on the two hallmark patterns of NREM sleep, slow oscillations and sleep spindles. These analyses entailed automated detection of discrete slow oscillation and spindle events, and calculation of the average characteristics of these events across stimulation conditions (STIM and SHAM). Given the forehead derivations adopted in this study, only frontally occurring slow spindles could be assessed [59].

**Figure 4:**
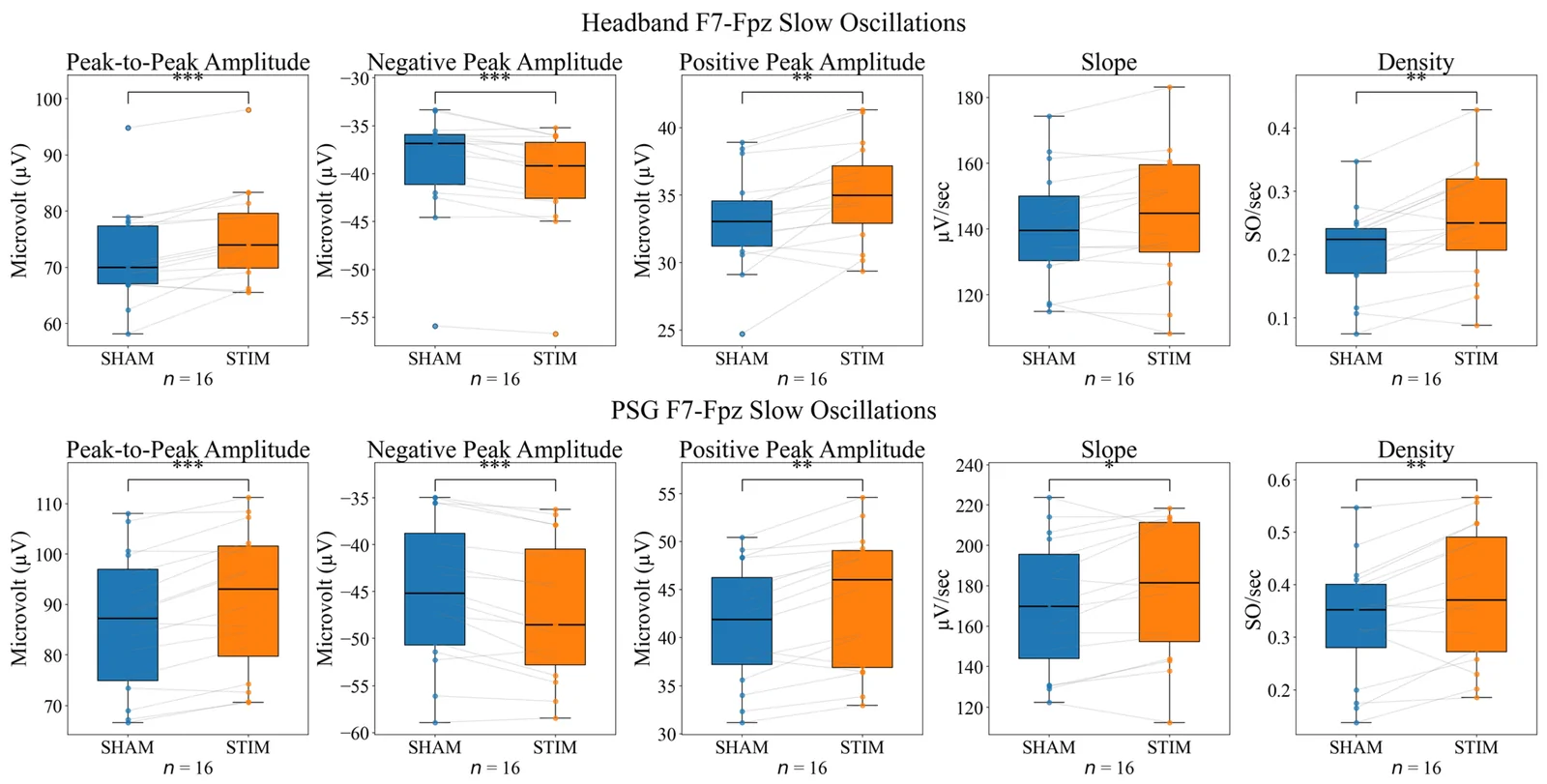
Slow-wave characteristics for STIM (orange) and SHAM (blue). The first row shows the headband F7-Fpz and the second row shows the PSG F7-Fpz (*n* = 16). Panels show peak-to-peak amplitude, negative-peak amplitude, and positive-peak amplitude (µV), slope (µV/s), and density (slow oscillations per second). * *p* < 0.05, ** *p* < 0.01, *** *p* < 0.001.

For slow oscillations, STIM increased peak-to-peak amplitude, negative-peak amplitude, positive-peak amplitude, and slow-wave density relative to SHAM in both the headband F7-Fpz and PSG F7-Fpz (Table 5; Figure 4). In PSG F7-Fpz, STIM also increased the slow oscillation slope relative to SHAM, while this effect was not observed in the headband. Thus, both recordings showed evidence of enhanced slow oscillation activity, and it appears that these enhancements can be reliably assessed from the headband EEG, for most, but not all, slow oscillation characteristics.

**Figure 5:**
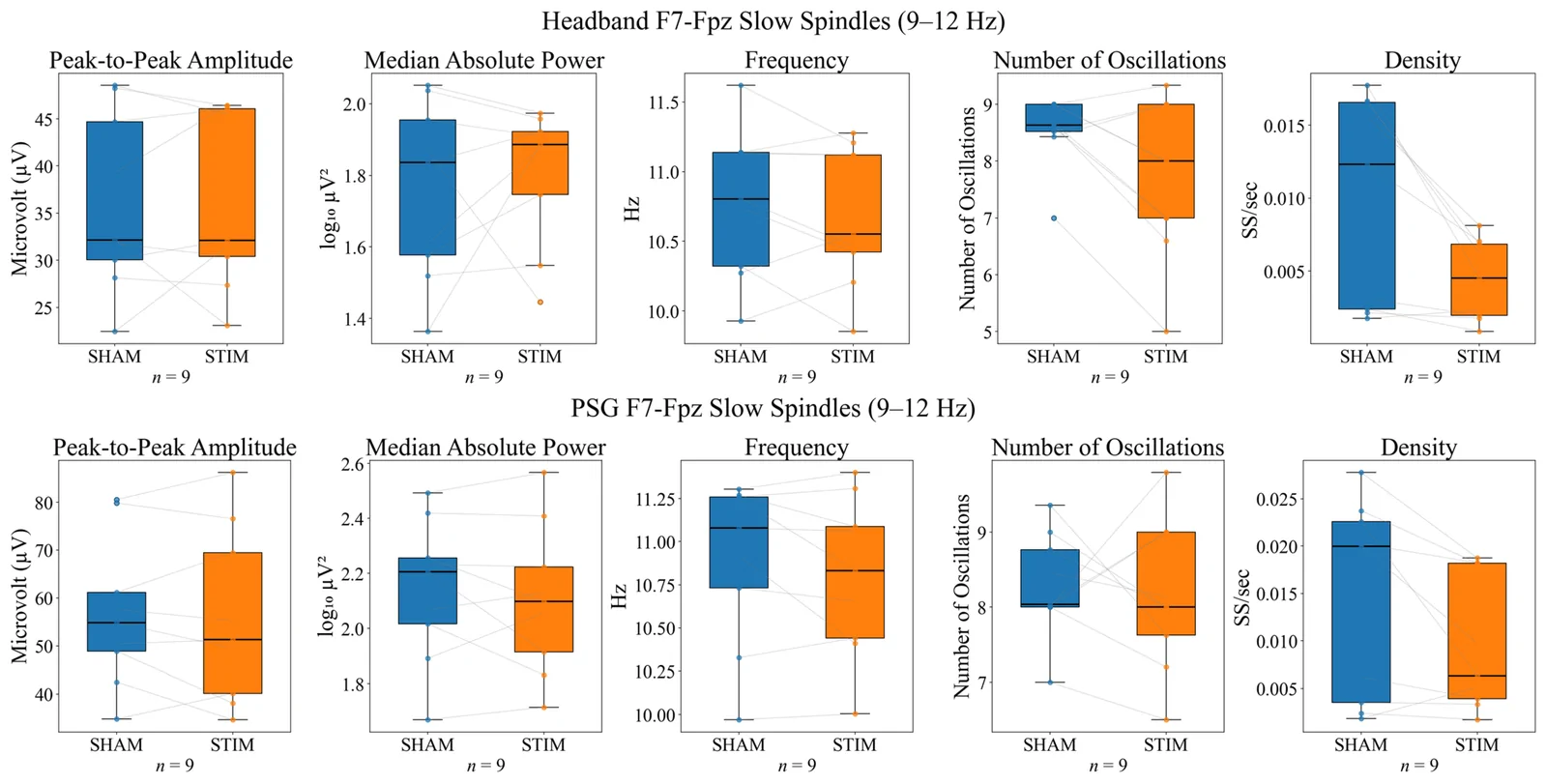
Slow-spindle characteristics for STIM (orange) and SHAM (blue). The first row shows the headband F7-Fpz and the second row shows the PSG F7-Fpz (*n* = 9). Panels show peak-to-peak amplitude (µV), median absolute power (log10 µV^2^), frequency (Hz), number of oscillations as part of the spindle, and density (spindles per second).

Slow spindles could be detected in only 9 of the 16 participants; subsequent slow-spindle outcomes were therefore based on this subsample (Table 6; Figure 5) and were therefore considered exploratory. Spindle density showed a reduction in the STIM condition relative to SHAM in both the headband F7-Fpz (*p* = 0.012) and PSG F7-Fpz (*p* = 0.047) before multiple comparison correction, but reduced to trend-level significance for the headband (*p* = 0.062) and non-significant in PSG after correction. In both the headband F7-Fpz and the PSG F7-Fpz data, no differences were found for peak-to-peak amplitude, median absolute power, frequency, or number of oscillations after FDR correction. The number of oscillations per spindle showed a trend-level decrease in STIM relative to SHAM in the headband (*p* = 0.098) but was non-significant in the PSG.

Collectively, the concordant enhancement of slow oscillations in the headband and PSG recordings demonstrates that the headband can capture stimulation-induced modulation of this key NREM sleep hallmark. Slow-spindle density was numerically lower during stimulation; however, this should be interpreted cautiously given the limited number of participants with detectable spindles and the loss of significance after correction.

## 4. Discussion

In this study, we investigated whether modeling-based closed-loop neurostimulation (M-CLNS) could be implemented using EEG acquired from a wireless EEG headband with forehead electrode derivations. Three principal findings support the technical feasibility of this approach. First, the headband-derived signal enabled individual acoustic stimuli to be targeted to the intended phase of ongoing slow oscillations. Second, stimulation elicited immediate SO-like responses in stimulus-locked analyses. Third, stimulation was associated with changes across stimulated slow-wave sleep, including increased SO and delta power and greater SO density and amplitude. These stimulation-related effects were independently confirmed through simultaneously recorded PSG and were also detectable in the headband-derived EEG. Despite signal-quality limitations in multiple participants, the findings demonstrate that a wireless EEG headband with forehead derivations can, in principle, be used to administer M-CLNS and to capture EEG responses to acoustic stimulation.

The correspondence between the wireless EEG headband and PSG findings is notable despite substantial differences between the two recording modalities. The forehead-derived headband recordings had approximately 40% lower amplitudes compared to conventional referenced EEG, visually contained more artifacts, and required exclusion of 5 of 26 participants due to data quality issues. The corresponding PSG recordings from these participants did not require exclusion for data quality reasons. Nevertheless, the direction and temporal profile of both the immediate stimulus-locked responses and the longer-term effects of stimulation were generally consistent across the two modalities. This similarity suggests that differences in electrode derivation, signal amplitude, and recording quality did not prevent the headband from providing physiologically meaningful input for closed-loop control or from capturing stimulation-related EEG changes.

A relevant feature of M-CLNS in this context is that its signal modeling does not depend on a fixed absolute-amplitude threshold. This may be advantageous for recordings obtained with wireless EEG headbands, in which signal amplitudes can differ substantially from that of conventional PSG because of electrode placement, referencing, and acquisition hardware. Other approaches, including amplitude-based detectors, phase-locked loops, and phase-vocoder techniques, may be more sensitive to reduced signal amplitude or signal-to-noise ratio [60]. The ability to operate on the lower-amplitude forehead-derived signals observed in the present study therefore represents an important technical property for implementation in an EEG headband.

The results provide evidence that M-CLNS using EEG acquired from a wireless headband can influence sleep activity over longer timescales. Acoustic stimulation increased SO and delta power as well as SO density and amplitude across stimulated SWS. These findings corroborate our previous observation of overall sleep deepening with PSG-based M-CLNS during a daytime nap [41] and extend it to an overnight stimulation protocol. The present study additionally demonstrates that these changes can be induced and detected using forehead-derived EEG from the wireless headband, supporting its potential use not only to facilitate stimulation but also to evaluate its physiological effects. In line with this previous nap study, a similar, non-significant but notable, reduction of sigma power was observed [41]. These trends may reflect an inverse relation between NREM sleep depth and spindle genesis [41,61,62] or differences in arousal sensitivity.

Sleep enhancement has previously been investigated using consumer-oriented wireless EEG headbands with more conventional frontal-to-mastoid derivations. Several studies used short rhythmic stimulation trains delivered through the SmartSleep Headband (Philips Healthcare, Amsterdam, The Netherlands) [63,64,65,66], whereas others used longer rhythmic trains with the DREEM headband (Rythm SAS, Paris, France) [67,68]. These studies primarily examined effects during ongoing rhythmic stimulation [63,64,65,66] or within the first seconds after stimulus onset [67,68]. They generally reported immediate SO-like event-related responses, increased stimulus-locked delta power [67,68], or increased delta power while rhythmic stimulation was ongoing [63,64,65,66]. However, they did not establish whether individual phase-targeted stimuli produce changes that persist across stimulated SWS. Moreover, the effects of rhythmic stimulus trains have been reported to attenuate with successive stimuli [37]. Differences in stimulation pattern, targeting method, and analysis window limit direct comparisons between these studies and the present protocol.

More durable changes have been reported in studies using individually SO-phase-targeted stimuli. Implementations based on versions of the Mobile Health Systems Lab Sleep Band (MHSL-SB; ETH Zurich, Zurich, Switzerland) [69] increased low-frequency slow-wave activity (0.75–1.25 Hz) on stimulation nights compared with sham nights in older adults [70] and, more recently, in a proof-of-concept study involving patients with Parkinson’s disease [71]. Another study in healthy young adults reported broader increases in delta–theta and sigma power during stimulation compared with sham nights [72]. Together with the present results, these findings support the capacity of individual phase-targeted acoustic stimuli to enhance slow-wave activity. The current study extends this literature by evaluating the forehead-derived signal from a wireless EEG headband against simultaneous PSG and by examining both immediate stimulus-locked responses and changes across stimulated SWS.

Several limitations should be considered. First, the study included healthy young individuals, who were selected for this initial technical feasibility assessment. The results may not generalize to older individuals or clinical populations, in whom SO amplitude, sleep architecture, artifact prevalence, and stimulation responses may differ. Second, the single-night, within-subject block design cannot, despite the insertion of washout blocks, exclude carryover between stimulation and sham periods or compensatory changes in sleep depth during sham blocks. Confirmation in randomized multi-night designs with independent stimulation and sham nights is therefore required.

Third, recordings were conducted in a controlled laboratory environment with simultaneous PSG. While this design enabled direct validation of headband findings against a gold-standard recording, it does not reflect standalone use of the wireless EEG headband. Fully independent use would require development of automated real-time sleep-stage scoring to restrict stimulation to selected NREM sleep stages, reliable self-application of the EEG headband without technician assistance, and robust as well as secure data transfer from the home environment to a secure server. To verify standalone use, the complete system would need to be evaluated under unsupervised home conditions. Environmental noise, device placement, movement and wireless signal interruptions could all affect signal quality and stimulation performance.

Future studies should consequently assess the system during repeated, unsupervised home recordings and examine automated signal-quality monitoring, artifact handling, electrode placement reliability, and recovery from data or connection loss. Evaluation in older adults and relevant clinical populations will be particularly informative because these groups commonly exhibit reduced SO amplitude and density [14,73]. Such studies should establish whether the technical performance and physiological effects observed here are retained under lower-amplitude and more variable recording conditions.

Overall, the findings demonstrate that the essential functions of PSG-based M-CLNS can be implemented using a wireless EEG headband with forehead electrode derivations under controlled laboratory conditions. The headband-derived signal supported real-time SO-phase targeting and captured stimulation-related EEG responses in the slow oscillations and delta domain that were also observed in simultaneous PSG. These results provide a technical basis for further developments toward a standalone M-CLNS system for repeated use in home and clinical environments. Such systems could eventually provide a scalable therapeutic approach for SWS disturbances in numerous conditions, including sleep, psychiatric, and neurological disorders [7,8,9,10,11,12,13,74].

## 5. Conclusions

M-CLNS implemented using EEG acquired from a wireless headband enabled real-time targeting of acoustic stimuli to SOs and produced both immediate stimulus-locked and longer-term increases in slow-wave activity. Phase-targeting performance and the resulting stimulation effects in the headband-derived EEG were corroborated by simultaneous PSG, indicating that the wireless EEG headband can serve as the sensing input for M-CLNS and can measure the resulting electrophysiological responses. These findings support the feasibility of closed-loop acoustic stimulation with a wireless EEG headband and set the stage for multi-night evaluations in home and clinical settings.

## 6. Patents

Lucia Talamini is an inventor of the patented modeling-based closed-loop neurostimulation method utilized in this study.

## Figures and Tables

**Table 1:** Participant exclusion overview.

	*n*
**Enrolled**	**26**
**Excluded due to EEG headband signal quality**	**5**
Poor signal quality >98% of F7–Fpz recording	2
Quality issues during stimulation period	3
**Excluded due to procedural or sleep criteria**	**5**
Did not complete the sleep interval	1
No signs of slow-wave sleep	1
Insufficient spontaneous slow-wave sleep continuity prior to stimulation	2
Computer issues	1
**Included in final analyses**	**16**

*n* = number of participants. Indented rows give the reason within each exclusion category.

**Table 2:** Sleep architecture based on PSG signals.

	Total Sleep Time (min)	NREM1 (% TST)	NREM2 (% TST)	NREM3 (% TST)	REM (% TST)
**Mean**	482.69	4.52	48.69	22.68	24.12
**SD**	34.35	2.87	6.61	6.47	4.56

TST = Total sleep time. min = minutes. % TST = percentage of the total sleep time. NREM = non-rapid eye movement. REM = rapid eye movement. SD = standard deviation.

**Table 3:** Significant time clusters (*p* < 0.05; in ms) where M-CLNS with acoustic stimulation enhanced SO activity for headband F7-Fpz and PSG F7-Fpz signals, along with the percentage overlap of these significant time ranges between the two signals. The intermediate headband cluster was at trend level (*p* = 0.050).

Headband F7-Fpz			PSG F7-Fpz			Headband-PSG Overlap
Start	End		Start	End		
Time (ms)	Time (ms)	*p*	Time (ms)	Time (ms)	*p*	Percentage (%)
98	730	<0.001	332	660	0.019	51.9
852	1129	0.05	793	1184	<0.001	100
1387	1934	0.003	1395	1762	0.011	67.09

ms = milliseconds. PSG = polysomnography.

**Table 4:** Means, standard deviations, paired samples *t*-test and Wilcoxon signed-rank statistics, effect sizes, confidence intervals, and percentage difference comparing frequency bin Welch spectral power between STIM and SHAM conditions for both headband F7-Fpz and PSG F7-Fpz signals. Only frequency bins showing statistically significant differences are included in the table.

	**Headband F7-Fpz**									
	**STIM**		**SHAM**		**Diff**				**95% CI**	
**Hz**	**Mean**	**SD**	**Mean**	**SD**	**(%)**	***t(13)/W***	***p***	***d/r***	**Low**	**High**
0.75	129.71	49.64	108.37	49.82	19.69	5.59	0.006	1.49	13.10	29.59
1.00	110.85	32.38	88.87	27.22	24.73	6.55	0.003	1.75	14.73	29.23
1.25	83.45	24.90	68.84	20.02	21.22	1	0.007	0.99	9.35	19.87
1.50	55.42	18.28	45.47	14.43	21.87	5.07	0.007	1.36	5.71	14.18
1.75	35.40	12.81	29.08	10.72	21.75	4.35	0.012	1.16	3.18	9.46
2.00	22.49	8.81	19.00	8.29	18.36	3.81	0.025	1.02	1.51	5.47
2.25	14.99	6.24	12.97	6.08	15.58	3.53	0.032	0.94	0.785	3.25
2.50	10.52	4.64	9.20	4.15	14.34	4.41	0.012	1.18	0.6725	1.96
2.75	7.65	3.32	6.64	2.80	15.33	4.81	0.007	1.29	0.5606	1.47
3.00	5.84	2.27	5.06	2.04	15.48	4.30	0.012	1.15	0.3897	1.18
3.25	4.67	1.63	4.11	1.59	13.64	3.96	0.021	1.06	0.2546	0.8654
3.50	3.86	1.31	3.41	1.26	13.05	5.35	0.006	1.43	0.2654	0.6254
3.75	3.30	1.12	2.89	1.05	14.40	4.91	0.007	1.31	0.2328	0.5984
4.00	2.85	0.9719	2.48	0.9419	14.92	3.66	0.029	0.98	0.1518	0.5881
4.25	2.40	0.8357	2.12	0.801	13.51	3.55	0.032	0.95	0.1121	0.4598
4.50	1.99	0.6944	1.79	0.6233	11.33	3.68	0.029	0.98	0.0837	0.3218
4.75	1.71	0.5875	1.55	0.5083	10.42	3.49	0.033	0.93	0.0613	0.2611
5.00	1.52	0.5245	1.37	0.4526	10.61	3.46	0.033	0.93	0.0548	0.2365
	**PSG F7-Fpz**									
	**STIM**		**SHAM**		**Diff**				**95% CI**	
**Hz**	**Mean**	**SD**	**Mean**	**SD**	**(%)**	***t(13)/W***	***p***	***d/r***	**low**	**high**
0.75	196.25	91.26	166.76	74.61	17.68	4.25	0.021	1.14	14.50	44.47
1.00	215.27	105.17	170.85	81.21	26.00	5.10	0.021	1.36	25.61	63.23
1.25	172.10	91.83	137.04	70.69	25.58	4.52	0.021	1.21	18.30	51.81
1.50	110.92	57.48	88.24	44.29	25.70	4.50	0.021	1.20	11.79	33.56
1.75	68.18	33.91	53.65	25.66	27.08	4.86	0.021	1.30	8.07	20.99
2.00	42.20	21.31	34.12	16.71	23.69	4.17	0.021	1.11	3.89	12.27
2.25							ns			
2.50							ns			
2.75	13.56	6.89	11.47	5.23	18.24	5	0.021	0.95	0.7408	3.44
3.00							ns			
3.25	8.14	3.64	7.12	3.10	14.29	3.66	0.040	0.98	0.4174	1.62
3.50	6.62	2.87	5.91	2.51	11.91	4.15	0.021	1.11	0.338	1.07
3.75	5.67	2.56	4.97	2.09	14.00	3.62	0.040	0.97	0.2807	1.11
4.00	5.02	2.29	4.32	1.88	16.16	3.68	0.040	0.98	0.2883	1.11
4.25							ns			
4.50							ns			
4.75							ns			
5.00							ns			

PSG = polysomnography; STIM = acoustic stimulation; SHAM = muted stimulation; SD = standard deviation; CI = confidence interval; Hz = hertz; W = Wilcoxon signed-rank statistic; d/r = effect size; ns = non-significant. Diff = percentage difference between STIM and SHAM. Positive difference scores indicate higher spectral power in STIM compared to SHAM.

**Table 5:** Slow wave characteristics. Means, standard deviations, paired samples *t*-test statistics, Wilcoxon signed-rank test statistics, effect sizes, and 95% confidence intervals, comparing slow wave characteristics between STIM and SHAM conditions for both headband F7-Fpz and PSG F7-Fpz signals.

	**Headband F7-Fpz**								
	**STIM**		**SHAM**					**95% CI**	
**Characteristic**	**Mean**	**SD**	**Mean**	**SD**	***t(15)/W***	***p***	***d/r***	**Low**	**High**
PTP Amplitude	75.51	8.42	72.07	8.47	4.62	<0.001	1.15	1.85	5.02
NP Amplitude	−40.43	5.39	−38.99	5.55	−4.72	<0.001	−1.18	−2.08	−0.787
PP Amplitude	35.08	3.65	33.08	3.63	3.79	0.002	0.95	0.875	3.12
Slope	275.91	37.27	267.31	31.5	2.44	0.057	0.61	1.09	16.1
Density	0.252	0.088	0.205	0.069	127	0.002	1.17	0.025	0.068
	**PSG F7-Fpz**								
	**STIM**		**SHAM**					**95% CI**	
**Characteristic**	**Mean**	**SD**	**Mean**	**SD**	***t(15)/W***	***p***	***d/r***	**low**	**high**
PTP Amplitude	90.87	14.06	86.37	13.58	5.36	<0.001	1.34	2.71	6.29
NP Amplitude	−47.05	7.4	−45.02	7.62	−4.66	<0.001	−1.17	−2.95	−1.1
PP Amplitude	43.82	7.02	41.34	6.22	128	0.001	1.2	1.38	3.58
Slope	355.32	67.71	337.03	62.72	2.82	0.016	0.71	4.48	32.1
Density	0.381	0.129	0.332	0.115	3.63	0.003	0.91	0.02	0.077

PSG = polysomnography; STIM = acoustic stimulation; SHAM = muted stimulation; SD = standard deviation; CI = confidence interval; W = Wilcoxon signed-rank statistic; d/r = effect size; PTP = peak-to-peak; NP = negative peak; PP = positive peak. Slope reflects the slope between the negative peak and zero crossing in µV per second. Density reflects the number of detected slow waves per second.

**Table 6:** Spindle characteristics. Means, standard deviations, paired samples *t*-test statistics, effect sizes, and 95% confidence intervals, comparing slow sleep spindle characteristics between STIM and SHAM conditions for both headband F7-Fpz and PSG F7-Fpz signals.

	**Headband F7-Fpz**								
	**STIM**		**SHAM**					**95% CI**	
**Characteristic**	**Mean**	**SD**	**Mean**	**SD**	***t(8)***	***p***	***d/r***	**Low**	**High**
PTP Amplitude	36.53	9.44	36.15	9.4	0.21	0.841	0.07	−3.79	4.54
Median Absolute Power	1.81	0.189	1.76	0.247	0.57	0.729	0.19	−0.151	0.251
Frequency	10.69	0.507	10.79	0.534	−0.98	0.591	−0.33	−0.324	0.13
Oscillations	7.66	1.4	8.54	0.617	−2.46	0.098	−0.82	−1.7	−0.057
Density	0.004	0.003	0.01	0.007	−3.21	0.062	−1.07	−0.009	−0.002
	**PSG F7-Fpz**								
	**STIM**		**SHAM**					**95% CI**	
**Characteristic**	**Mean**	**SD**	**Mean**	**SD**	***t(8)***	***p***	***d/r***	**low**	**high**
PTP Amplitude	55.68	18.07	56.74	15.46	−0.49	0.704	−0.16	−6.11	3.99
Median Absolute Power	2.11	0.271	2.14	0.258	−0.69	0.704	−0.23	−0.147	0.079
Frequency	10.8	0.462	10.9	0.475	−1.34	0.539	−0.45	−0.274	0.072
Oscillations	8.14	1.01	8.29	0.698	−0.39	0.704	−0.13	−1.03	0.733
Density	0.01	0.007	0.014	0.011	−2.35	0.234	−0.78	−0.01	−0.0001

PSG = polysomnography; STIM = acoustic stimulation; SHAM = muted stimulation; SD = standard deviation; CI = confidence interval; d/r = effect size; PTP = peak-to-peak. Density reflects the number of detected slow sleep spindles per second. Oscillations reflect the number of positive peaks in the spindle.

## Data Availability

The data supporting the conclusions of this article will be made available by the authors on reasonable request.

## Supplementary Materials

The following supporting information can be downloaded at: https://www.mdpi.com/article/10.3390/s26206528/s1, Table S1: Means, standard deviations, paired samples *t*-test and Wilcoxon signed-rank statistics, effect sizes, confidence intervals, and percentage difference comparing frequency bin Welch spectral power between STIM and SHAM conditions for both PSG F7-Fpz and headband F7-Fpz signals.
