# Supplementary material for: Closed-Loop Acoustic Stimulation of Slow Oscillations Using a Wireless EEG Headband: A Polysomnography-Validated Feasibility Study
Source: Sensors (Basel). 2026 Oct 15;26(20):6528. doi: 10.3390/s26206528 (PMC13709315; doi:10.3390/s26206528)
Supplement: Supplementary file 1 [file sensors-26-06528-s001.zip › sensors-4575552-supplementary.pdf]

Supplementary results

Table S1. Means, standard deviations, paired samples t-test and Wilcoxon signed-rank statistics, effect sizes, confidence intervals, and percentage difference comparing frequency bin Welch spectral power between STIM and SHAM conditions for both PSG F7-Fpz and headband F7-Fpz signals.

	**Headband F7-Fpz**										**PSG F7-Fpz**									
	**STIM**		**SHAM**		**Diff**				**95% CI**		**STIM**		**SHAM**		**Diff**				**95% CI**	
**Hz**	**Mean**	**SD**	**Mean**	**SD**	**(%)**	**t(13)/W**	**p**	**d/r**	**low**	**high**	**Mean**	**SD**	**Mean**	**SD**	**(%)**	**t(13)/W**	**p**	**d/r**	**low**	**high**
0.25	123.36	103.19	127.08	115.99	-2.93	-0.36	.866	-0.10	-25.99	18.55	128.25	133.25	139.07	134.16	-7.78	36	.702	0.66	-26.54	4.91
0.50	170.82	122.17	164.06	130.21	4.12	0.62	.706	0.16	-16.99	30.52	191.67	149.91	195.52	149.08	-1.97	50	.980	0.52	-22.62	14.92
0.75	129.71	49.64	108.37	49.82	19.69	5.59	**.006**	1.49	13.10	29.59	196.25	91.26	166.76	74.61	17.68	4.25	**.021**	1.14	14.50	44.47
1.00	110.85	32.38	88.87	27.22	24.73	6.55	**.003**	1.75	14.73	29.23	215.27	105.17	170.85	81.21	26.00	5.10	**.021**	1.36	25.61	63.23
1.25	83.45	24.90	68.84	20.02	21.22	1	**.007**	0.99	9.35	19.87	172.10	91.83	137.04	70.69	25.58	4.52	**.021**	1.21	18.30	51.81
1.50	55.42	18.28	45.47	14.43	21.87	5.07	**.007**	1.36	5.71	14.18	110.92	57.48	88.24	44.29	25.70	4.50	**.021**	1.20	11.79	33.56
1.75	35.40	12.81	29.08	10.72	21.75	4.35	**.012**	1.16	3.18	9.46	68.18	33.91	53.65	25.66	27.08	4.86	**.021**	1.30	8.07	20.99
2.00	22.49	8.81	19.00	8.29	18.36	3.81	**.025**	1.02	1.51	5.47	42.20	21.31	34.12	16.71	23.69	4.17	**.021**	1.11	3.89	12.27
2.25	14.99	6.24	12.97	6.08	15.58	3.53	**.032**	0.94	0.7850	3.25	27.40	14.15	22.87	11.20	19.80	2.84	.114	0.76	1.09	7.97
2.50	10.52	4.64	9.20	4.15	14.34	4.41	**.012**	1.18	0.6725	1.96	18.83	9.65	15.98	7.46	17.81	2.85	.114	0.76	0.6874	5.01
2.75	7.65	3.32	6.64	2.80	15.33	4.81	**.007**	1.29	0.5606	1.47	13.56	6.89	11.47	5.23	18.24	5	**.021**	0.95	0.7408	3.44
3.00	5.84	2.27	5.06	2.04	15.48	4.30	**.012**	1.15	0.3897	1.18	10.28	5.04	8.74	3.90	17.61	3.22	.073	0.86	0.5057	2.57
3.25	4.67	1.63	4.11	1.59	13.64	3.96	**.021**	1.06	0.2546	0.8654	8.14	3.64	7.12	3.10	14.29	3.66	**.040**	0.98	0.4174	1.62
3.50	3.86	1.31	3.41	1.26	13.05	5.35	**.006**	1.43	0.2654	0.6254	6.62	2.87	5.91	2.51	11.91	4.15	**.021**	1.11	0.3380	1.07
3.75	3.30	1.12	2.89	1.05	14.40	4.91	**.007**	1.31	0.2328	0.5984	5.67	2.56	4.97	2.09	14.00	3.62	**.040**	0.97	0.2807	1.11
4.00	2.85	0.9719	2.48	0.9419	14.92	3.66	**.029**	0.98	0.1518	0.5881	5.02	2.29	4.32	1.88	16.16	3.68	**.040**	0.98	0.2883	1.11
4.25	2.40	0.8357	2.12	0.8010	13.51	3.55	**.032**	0.95	0.1121	0.4598	4.32	2.03	3.75	1.64	15.14	3.31	.066	0.88	0.1968	0.9388
4.50	1.99	0.6944	1.79	0.6233	11.33	3.68	**.029**	0.98	0.0837	0.3218	3.63	1.77	3.20	1.35	13.56	2.64	.130	0.71	0.0789	0.7882
4.75	1.71	0.5875	1.55	0.5083	10.42	3.49	**.033**	0.93	0.0613	0.2611	3.12	1.50	2.74	1.12	13.94	2.73	.114	0.73	0.0797	0.6843
5.00	1.52	0.5245	1.37	0.4526	10.61	3.46	**.033**	0.93	0.0548	0.2365	2.79	1.36	2.43	0.99	14.79	2.73	.114	0.73	0.0754	0.6439
5.25	1.33	0.4634	1.22	0.4165	9.11	3.15	.056	0.84	0.0350	0.1877	2.49	1.27	2.21	0.93	12.86	14	.114	0.87	0.0295	0.5380

5.50	1.16	0.3899	1.10	0.3987	4.59	2.37	.228	0.63	0.0044	0.0970	2.18	1.07	1.98	0.84	9.63	15	.114	0.86	0.0157	0.3666
5.75	1.05	0.3578	1.02	0.3757	2.90	1.43	.526	0.38	-0.0152	0.0743	1.95	0.9164	1.82	0.77	7.35	19	.198	0.82	0.0083	0.2590
6.00	0.9734	0.3460	0.9268	0.3495	5.02	2.05	.338	0.55	-0.0025	0.0956	1.80	0.8876	1.69	0.76	6.52	1.67	.451	0.45	-0.0325	0.2523
6.25	0.8869	0.3407	0.8599	0.3393	3.14	1.30	.526	0.35	-0.0179	0.0719	1.65	0.8682	1.58	0.75	4.42	1.08	.662	0.29	-0.0691	0.2083
6.50	0.8082	0.3402	0.8079	0.3591	0.04	0.02	1.000	0.00	-0.0405	0.0411	1.50	0.8042	1.49	0.79	0.62	0.21	.946	0.06	-0.0859	0.1045
6.75	0.7594	0.3496	0.7449	0.3705	1.95	0.83	.634	0.22	-0.0235	0.0525	1.39	0.7628	1.39	0.77	0.56	0.18	.946	0.05	-0.0837	0.0992
7.00	0.7230	0.3627	0.6906	0.3594	4.68	3.09	.060	0.83	0.0097	0.0549	1.31	0.7487	1.29	0.73	1.90	0.72	.789	0.19	-0.0494	0.0985
7.25	0.6690	0.3544	0.6553	0.3652	2.08	1.33	.526	0.35	-0.0086	0.0359	1.23	0.7341	1.23	0.75	-0.59	-0.27	.923	-0.07	-0.0650	0.0505
7.50	0.6109	0.3386	0.6141	0.3639	-0.53	-0.22	.908	-0.06	-0.0352	0.0287	1.15	0.7387	1.16	0.73	-0.85	-0.33	.895	-0.09	-0.0747	0.0549
7.75	0.5590	0.3274	0.5563	0.3418	0.49	0.18	.929	0.05	-0.0295	0.0349	1.06	0.7259	1.06	0.70	0.00	0.00	1.000	0.00	-0.0727	0.0728
8.00	0.5188	0.3210	0.5021	0.3065	3.34	1.51	.513	0.40	-0.0072	0.0407	0.9874	0.6973	0.9749	0.66	1.29	49	.946	0.53	-0.0428	0.0679
8.25	0.4795	0.3019	0.4611	0.2773	3.99	1.51	.513	0.40	-0.0079	0.0447	0.9220	0.6682	0.8911	0.60	3.47	43	.808	0.59	-0.0383	0.1001
8.50	0.4369	0.2762	0.4272	0.2643	2.28	0.73	.667	0.19	-0.0193	0.0387	0.8595	0.6455	0.8387	0.60	2.49	43	.808	0.59	-0.0486	0.0903
8.75	0.4069	0.2691	0.4042	0.2561	0.66	48	.908	0.54	-0.0305	0.0359	0.8175	0.6653	0.8118	0.64	0.70	46	.870	0.56	-0.0570	0.0683
9.00	0.3896	0.2706	0.3921	0.2586	-0.63	-0.17	.929	-0.05	-0.0339	0.0289	0.8043	0.7364	0.8007	0.69	0.45	0.13	.980	0.03	-0.0560	0.0631
9.25	0.3860	0.2844	0.3897	0.2771	-0.97	-0.26	.908	-0.07	-0.0346	0.0271	0.8316	0.8638	0.8217	0.79	1.20	51	1.000	0.51	-0.0556	0.0754
9.50	0.3926	0.3087	0.4043	0.3075	-2.89	-0.63	.705	-0.17	-0.0515	0.0281	0.8856	1.0137	0.8954	0.93	-1.09	40	.789	0.62	-0.0959	0.0764
9.75	0.4007	0.3235	0.4061	0.3079	-1.32	38	.634	0.64	-0.0512	0.0404	0.9356	1.0964	0.9480	1.00	-1.31	32	.606	0.70	-0.1156	0.0908
10.00	0.3946	0.2979	0.3894	0.2799	1.34	52	1.000	0.50	-0.0248	0.0353	0.9142	0.9913	0.9342	0.9388	-2.14	32	.606	0.70	-0.0881	0.0481
10.25	0.3760	0.2576	0.3875	0.2640	-2.96	-1.14	.593	-0.31	-0.0331	0.0102	0.8476	0.8273	0.9205	0.8681	-7.93	-2.81	.114	-0.75	-0.1290	-0.0169
10.50	0.3557	0.2369	0.3892	0.2662	-8.62	32	.526	0.70	-0.0689	0.0018	0.8069	0.7873	0.9101	0.8624	-11.34	14	.114	0.87	-0.1975	-0.0089
10.75	0.3378	0.2273	0.3825	0.2790	-11.68	27	.502	0.74	-0.0952	0.0058	0.7817	0.7900	0.8863	0.8884	-11.80	25	.352	0.76	-0.2266	0.0174
11.00	0.3313	0.2254	0.3807	0.2933	-12.98	31	.526	0.70	-0.1078	0.0089	0.7560	0.7723	0.8646	0.8806	-12.56	25	.352	0.76	-0.2367	0.0195
11.25	0.3316	0.2234	0.3821	0.3003	-13.23	22	.338	0.79	-0.1071	0.0060	0.7303	0.7108	0.8411	0.8325	-13.18	18	.172	0.83	-0.2375	0.0158
11.50	0.3271	0.2167	0.3767	0.3003	-13.16	23	.338	0.78	-0.1065	0.0073	0.7083	0.6385	0.8038	0.7630	-11.88	24	.343	0.77	-0.2207	0.0297
11.75	0.3180	0.2224	0.3595	0.2905	-11.54	29	.513	0.72	-0.0885	0.0055	0.6826	0.6141	0.7467	0.6810	-8.58	-1.49	.549	-0.40	-0.1573	0.0291
12.00	0.3002	0.2233	0.3249	0.2656	-7.60	31	.526	0.70	-0.0552	0.0059	0.6361	0.5734	0.6698	0.5884	-5.02	-1.23	.655	-0.33	-0.0927	0.0254
12.25	0.2657	0.1929	0.2819	0.2159	-5.76	-1.65	.502	-0.44	-0.0374	0.0050	0.5631	0.4722	0.5808	0.4630	-3.05	46	.870	0.56	-0.0698	0.0343

12.50	0.2244	0.1538	0.2420	0.1691	-7.28	-2.01	.338	-0.54	-0.0366	0.0013	0.4744	0.3621	0.4886	0.3440	-2.91	-0.61	.803	-0.16	-0.0643	0.0359
12.75	0.1900	0.1266	0.2021	0.1359	-5.96	27	.502	0.74	-0.0272	0.0031	0.3933	0.2774	0.4041	0.2636	-2.66	-0.60	.805	-0.16	-0.0493	0.0278
13.00	0.1612	0.1037	0.1615	0.1018	-0.20	-0.07	.987	-0.02	-0.0111	0.0104	0.3297	0.2188	0.3313	0.2001	-0.47	-0.11	.981	-0.03	-0.0308	0.0277
13.25	0.1371	0.0852	0.1296	0.0764	5.82	1.75	.477	0.47	-0.0018	0.0168	0.2812	0.1792	0.2687	0.1549	4.64	1.11	.662	0.30	-0.0118	0.0368
13.50	0.1163	0.0692	0.1100	0.0622	5.77	2.10	.338	0.56	-0.0002	0.0129	0.2434	0.1533	0.2247	0.1320	8.30	2.14	.278	0.57	-0.0002	0.0375
13.75	0.0993	0.0550	0.0964	0.0535	3.00	1.35	.526	0.36	-0.0017	0.0075	0.2105	0.1347	0.1972	0.1229	6.77	24	.343	0.77	-0.0026	0.0293
14.00	0.0859	0.0444	0.0836	0.0460	2.74	0.92	.634	0.24	-0.0031	0.0077	0.1812	0.1195	0.1735	0.1142	4.45	1.19	.662	0.32	-0.0063	0.0217
14.25	0.0739	0.0365	0.0723	0.0371	2.19	0.83	.634	0.22	-0.0025	0.0057	0.1552	0.1072	0.1508	0.0999	2.90	52	1.000	0.50	-0.0083	0.0170
14.50	0.0650	0.0316	0.0641	0.0310	1.40	0.53	.758	0.14	-0.0028	0.0046	0.1343	0.0950	0.1301	0.0846	3.20	51	1.000	0.51	-0.0078	0.0161
14.75	0.0588	0.0285	0.0575	0.0266	2.18	0.81	.634	0.22	-0.0021	0.0046	0.1188	0.0829	0.1122	0.0696	5.88	42	.803	0.60	-0.0059	0.0191
15.00	0.0540	0.0262	0.0518	0.0227	4.27	1.14	.593	0.31	-0.0020	0.0064	0.1043	0.0703	0.0980	0.0561	6.49	1.10	.662	0.29	-0.0062	0.0189
15.25	0.0502	0.0236	0.0477	0.0207	5.24	1.39	.526	0.37	-0.0014	0.0064	0.0915	0.0589	0.0877	0.0476	4.24	0.70	.789	0.19	-0.0077	0.0152
15.50	0.0466	0.0211	0.0438	0.0187	6.27	1.60	.504	0.43	-0.0010	0.0065	0.0826	0.0513	0.0789	0.0417	4.69	0.82	.789	0.22	-0.0061	0.0135
15.75	0.0431	0.0189	0.0404	0.0161	6.72	1.74	.477	0.46	-0.0007	0.0061	0.0741	0.0438	0.0710	0.0376	4.46	0.95	.732	0.25	-0.0041	0.0104
16.00	0.0402	0.0172	0.0382	0.0145	5.33	35	.609	0.67	-0.0009	0.0049	0.0667	0.0375	0.0651	0.0355	2.34	34	.662	0.68	-0.0037	0.0067
16.25	0.0381	0.0157	0.0366	0.0135	4.07	1.06	.627	0.28	-0.0016	0.0045	0.0618	0.0348	0.0599	0.0323	3.14	34	.662	0.68	-0.0037	0.0075
16.50	0.0359	0.0147	0.0352	0.0129	2.05	0.43	.814	0.12	-0.0029	0.0043	0.0576	0.0319	0.0555	0.0291	3.82	0.68	.791	0.18	-0.0046	0.0089
16.75	0.0337	0.0137	0.0333	0.0120	1.31	0.34	.875	0.09	-0.0023	0.0032	0.0541	0.0289	0.0522	0.0276	3.51	0.73	.789	0.19	-0.0036	0.0073
17.00	0.0323	0.0129	0.0315	0.0110	2.74	0.99	.630	0.26	-0.0010	0.0027	0.0516	0.0283	0.0499	0.0271	3.42	31	.577	0.70	-0.0031	0.0065
17.25	0.0316	0.0130	0.0297	0.0105	6.34	1.60	.504	0.43	-0.0007	0.0044	0.0488	0.0267	0.0475	0.0264	2.64	30	.549	0.71	-0.0040	0.0065
17.50	0.0309	0.0124	0.0279	0.0100	10.60	23	.338	0.78	0.0001	0.0058	0.0463	0.0242	0.0453	0.0251	2.16	31	.577	0.70	-0.0039	0.0058
17.75	0.0294	0.0114	0.0270	0.0097	8.96	30	.526	0.71	-0.0002	0.0050	0.0442	0.0239	0.0433	0.0241	2.01	30	.549	0.71	-0.0035	0.0052
18.00	0.0276	0.0108	0.0264	0.0093	4.76	1.13	.593	0.30	-0.0011	0.0036	0.0414	0.0238	0.0412	0.0226	0.45	38	.771	0.64	-0.0046	0.0050
18.25	0.0268	0.0111	0.0258	0.0084	3.89	0.77	.648	0.21	-0.0018	0.0038	0.0392	0.0232	0.0396	0.0200	-1.09	52	1.000	0.50	-0.0056	0.0047
18.50	0.0263	0.0110	0.0251	0.0081	4.73	0.83	.634	0.22	-0.0019	0.0043	0.0379	0.0219	0.0387	0.0192	-2.26	-0.44	.861	-0.12	-0.0052	0.0035
18.75	0.0255	0.0100	0.0237	0.0081	7.44	1.45	.526	0.39	-0.0009	0.0044	0.0366	0.0196	0.0373	0.0194	-2.00	52	1.000	0.50	-0.0039	0.0024
19.00	0.0247	0.0093	0.0227	0.0079	8.62	2.09	.338	0.56	-0.0001	0.0040	0.0356	0.0191	0.0358	0.0195	-0.42	46	.870	0.56	-0.0031	0.0028
19.25	0.0238	0.0091	0.0220	0.0076	7.96	1.83	.438	0.49	-0.0003	0.0038	0.0345	0.0189	0.0345	0.0187	0.05	43	.808	0.59	-0.0030	0.0030

19.50	0.0225	0.0087	0.0209	0.0069	7.63	1.50	.513	0.40	-0.0007	0.0039	0.0327	0.0174	0.0329	0.0172	-0.67	46	.870	0.56	-0.0037	0.0032
19.75	0.0213	0.0078	0.0202	0.0063	5.35	36	.630	0.66	-0.0007	0.0029	0.0311	0.0156	0.0316	0.0159	-1.60	49	.946	0.53	-0.0042	0.0032
20.00	0.0209	0.0075	0.0202	0.0062	3.30	0.92	.634	0.25	-0.0009	0.0022	0.0301	0.0146	0.0304	0.0149	-0.99	37	.732	0.65	-0.0040	0.0034
20.25	0.0205	0.0072	0.0200	0.0064	2.52	0.60	.706	0.16	-0.0013	0.0023	0.0291	0.0145	0.0296	0.0147	-1.46	44	.834	0.58	-0.0041	0.0033
20.50	0.0197	0.0069	0.0192	0.0062	2.75	0.63	.705	0.17	-0.0013	0.0023	0.0283	0.0150	0.0288	0.0141	-1.66	49	.946	0.53	-0.0042	0.0032
20.75	0.0191	0.0069	0.0186	0.0059	2.64	0.60	.706	0.16	-0.0013	0.0023	0.0273	0.0149	0.0283	0.0129	-3.32	40	.789	0.62	-0.0044	0.0025
21.00	0.0185	0.0068	0.0183	0.0060	1.01	0.28	.908	0.07	-0.0013	0.0016	0.0263	0.0138	0.0278	0.0126	-5.54	-1.15	.662	-0.31	-0.0044	0.0014
21.25	0.0183	0.0066	0.0178	0.0060	3.09	0.89	.634	0.24	-0.0008	0.0019	0.0255	0.0129	0.0271	0.0129	-5.76	35	.662	0.67	-0.0046	0.0015
21.50	0.0182	0.0065	0.0173	0.0056	5.35	1.33	.526	0.36	-0.0006	0.0024	0.0245	0.0120	0.0260	0.0126	-5.67	39	.789	0.63	-0.0047	0.0018
21.75	0.0179	0.0066	0.0171	0.0055	4.42	1.03	.630	0.28	-0.0008	0.0023	0.0237	0.0113	0.0249	0.0123	-4.73	47	.895	0.55	-0.0040	0.0017
22.00	0.0176	0.0066	0.0168	0.0057	4.78	1.10	.609	0.29	-0.0008	0.0024	0.0234	0.0112	0.0243	0.0119	-3.75	47	.895	0.55	-0.0033	0.0014
22.25	0.0170	0.0062	0.0164	0.0056	3.99	0.94	.634	0.25	-0.0009	0.0022	0.0227	0.0109	0.0234	0.0112	-3.06	-0.71	.789	-0.19	-0.0029	0.0015
22.50	0.0163	0.0059	0.0157	0.0054	3.84	0.90	.634	0.24	-0.0008	0.0020	0.0218	0.0106	0.0225	0.0107	-2.99	52	1.000	0.50	-0.0028	0.0014
22.75	0.0164	0.0059	0.0153	0.0051	6.96	1.63	.504	0.44	-0.0003	0.0025	0.0215	0.0105	0.0222	0.0104	-3.11	45	.861	0.57	-0.0033	0.0019
23.00	0.0165	0.0060	0.0154	0.0051	7.27	1.53	.513	0.41	-0.0005	0.0027	0.0212	0.0098	0.0222	0.0101	-4.67	41	.789	0.61	-0.0040	0.0019
23.25	0.0160	0.0062	0.0153	0.0052	4.49	51	.987	0.51	-0.0011	0.0024	0.0205	0.0091	0.0216	0.0096	-5.32	41	.789	0.61	-0.0041	0.0018
23.50	0.0153	0.0060	0.0148	0.0052	3.74	0.86	.634	0.23	-0.0008	0.0020	0.0202	0.0090	0.0209	0.0090	-3.14	-0.53	.828	-0.14	-0.0033	0.0020
23.75	0.0149	0.0058	0.0144	0.0051	3.76	1.03	.630	0.28	-0.0006	0.0017	0.0198	0.0087	0.0206	0.0087	-4.05	-0.79	.789	-0.21	-0.0031	0.0014
24.00	0.0146	0.0058	0.0139	0.0047	4.95	1.21	.575	0.32	-0.0005	0.0019	0.0191	0.0082	0.0203	0.0087	-6.12	34	.662	0.68	-0.0033	0.0008
24.25	0.0143	0.0058	0.0134	0.0044	7.05	1.43	.526	0.38	-0.0005	0.0024	0.0186	0.0075	0.0194	0.0080	-4.10	44	.834	0.58	-0.0026	0.0010
24.50	0.0139	0.0055	0.0131	0.0044	6.14	1.35	.526	0.36	-0.0005	0.0021	0.0178	0.0069	0.0186	0.0072	-4.52	44	.834	0.58	-0.0028	0.0011
24.75	0.0132	0.0049	0.0128	0.0045	2.99	0.86	.634	0.23	-0.0006	0.0013	0.0170	0.0063	0.0183	0.0070	-7.24	36	.702	0.66	-0.0036	0.0009
25.00	0.0125	0.0045	0.0126	0.0044	-0.82	-0.23	.908	-0.06	-0.0011	0.0008	0.0165	0.0057	0.0176	0.0066	-6.56	40	.789	0.62	-0.0032	0.0009
25.25	0.0124	0.0047	0.0124	0.0042	-0.12	-0.03	1.000	-0.01	-0.0010	0.0010	0.0162	0.0054	0.0171	0.0062	-5.23	42	.803	0.60	-0.0028	0.0010
25.50	0.0123	0.0049	0.0118	0.0039	4.18	0.97	.630	0.26	-0.0006	0.0016	0.0159	0.0052	0.0167	0.0060	-4.41	43	.808	0.59	-0.0026	0.0012
25.75	0.0120	0.0048	0.0114	0.0038	4.69	1.01	.630	0.27	-0.0006	0.0017	0.0154	0.0052	0.0159	0.0059	-3.29	52	1.000	0.50	-0.0025	0.0014
26.00	0.0115	0.0044	0.0112	0.0037	2.49	0.61	.706	0.16	-0.0007	0.0013	0.0148	0.0051	0.0155	0.0055	-4.46	-0.80	.789	-0.21	-0.0026	0.0012
26.25	0.0111	0.0040	0.0107	0.0035	2.98	0.81	.634	0.22	-0.0005	0.0012	0.0143	0.0048	0.0150	0.0052	-5.15	-0.96	.732	-0.26	-0.0025	0.0010

26.50	0.0111	0.0040	0.0105	0.0031	4.86	1.15	.593	0.31	-0.0004	0.0015	0.0141	0.0046	0.0146	0.0051	-3.93	51	1.000	0.51	-0.0025	0.0013
26.75	0.0110	0.0042	0.0106	0.0032	4.10	0.78	.648	0.21	-0.0008	0.0016	0.0140	0.0045	0.0145	0.0050	-4.06	46	.870	0.56	-0.0027	0.0015
27.00	0.0108	0.0042	0.0103	0.0031	5.03	0.97	.630	0.26	-0.0006	0.0017	0.0136	0.0045	0.0143	0.0049	-4.77	45	.861	0.57	-0.0026	0.0012
27.25	0.0106	0.0041	0.0100	0.0030	5.93	1.29	.526	0.35	-0.0004	0.0016	0.0133	0.0047	0.0139	0.0047	-4.37	-0.72	.789	-0.19	-0.0024	0.0012
27.50	0.0103	0.0039	0.0097	0.0029	5.85	1.35	.526	0.36	-0.0003	0.0015	0.0129	0.0048	0.0134	0.0048	-3.73	52	1.000	0.50	-0.0025	0.0015
27.75	0.0101	0.0038	0.0095	0.0028	6.20	1.22	.575	0.33	-0.0005	0.0016	0.0125	0.0045	0.0133	0.0048	-5.88	45	.861	0.57	-0.0028	0.0012
28.00	0.0098	0.0037	0.0093	0.0026	5.18	0.98	.630	0.26	-0.0006	0.0016	0.0123	0.0043	0.0132	0.0046	-7.27	38	.771	0.64	-0.0027	0.0008
28.25	0.0096	0.0035	0.0093	0.0026	2.93	0.63	.705	0.17	-0.0007	0.0012	0.0120	0.0039	0.0131	0.0044	-8.10	30	.549	0.71	-0.0026	0.0005
28.50	0.0096	0.0035	0.0095	0.0027	1.12	0.25	.908	0.07	-0.0008	0.0010	0.0118	0.0037	0.0127	0.0043	-6.74	42	.803	0.60	-0.0024	0.0007
28.75	0.0095	0.0034	0.0094	0.0027	1.10	0.22	.908	0.06	-0.0009	0.0011	0.0117	0.0040	0.0123	0.0042	-5.23	47	.895	0.55	-0.0023	0.0010
29.00	0.0092	0.0033	0.0092	0.0027	0.15	0.03	1.000	0.01	-0.0010	0.0011	0.0115	0.0042	0.0124	0.0042	-7.77	41	.789	0.61	-0.0026	0.0007
29.25	0.0088	0.0033	0.0089	0.0027	-0.81	41	.682	0.61	-0.0012	0.0011	0.0112	0.0042	0.0123	0.0042	-9.12	28	.498	0.73	-0.0028	0.0006
29.50	0.0087	0.0033	0.0086	0.0026	1.59	45	.814	0.57	-0.0010	0.0012	0.0111	0.0041	0.0119	0.0044	-6.92	39	.789	0.63	-0.0024	0.0008
29.75	0.0088	0.0035	0.0084	0.0026	3.72	50	.951	0.52	-0.0008	0.0015	0.0111	0.0044	0.0116	0.0044	-4.05	-0.62	.803	-0.17	-0.0021	0.0012
30.00	0.0086	0.0036	0.0085	0.0025	1.19	0.15	.939	0.04	-0.0014	0.0016	0.0109	0.0047	0.0114	0.0043	-4.90	-0.67	.791	-0.18	-0.0024	0.0012
30.25	0.0083	0.0034	0.0085	0.0025	-2.43	-0.33	.881	-0.09	-0.0016	0.0012	0.0106	0.0044	0.0113	0.0045	-5.80	49	.946	0.53	-0.0024	0.0011
30.50	0.0081	0.0030	0.0085	0.0025	-4.95	-0.88	.634	-0.24	-0.0014	0.0006	0.0104	0.0040	0.0111	0.0044	-6.79	48	.935	0.54	-0.0023	0.0008
30.75	0.0079	0.0028	0.0082	0.0025	-3.84	-0.79	.648	-0.21	-0.0012	0.0006	0.0101	0.0040	0.0109	0.0042	-8.11	35	.662	0.67	-0.0022	0.0004
31.00	0.0079	0.0029	0.0080	0.0024	-1.08	-0.23	.908	-0.06	-0.0009	0.0007	0.0100	0.0040	0.0108	0.0041	-7.17	-1.39	.577	-0.37	-0.0020	0.0004
31.25	0.0077	0.0029	0.0080	0.0024	-4.30	-0.85	.634	-0.23	-0.0012	0.0005	0.0100	0.0040	0.0108	0.0040	-7.22	-1.22	.655	-0.33	-0.0022	0.0006
31.50	0.0075	0.0026	0.0081	0.0025	-7.15	-1.58	.506	-0.42	-0.0014	0.0002	0.0099	0.0038	0.0108	0.0042	-8.16	46	.870	0.56	-0.0024	0.0007
31.75	0.0075	0.0026	0.0078	0.0024	-3.85	-0.98	.630	-0.26	-0.0010	0.0004	0.0096	0.0034	0.0105	0.0042	-8.04	40	.789	0.62	-0.0023	0.0006
32.00	0.0074	0.0027	0.0075	0.0023	-2.23	-0.48	.793	-0.13	-0.0009	0.0006	0.0095	0.0033	0.0103	0.0042	-8.47	35	.662	0.67	-0.0023	0.0006
32.25	0.0072	0.0027	0.0075	0.0023	-3.91	-0.71	.673	-0.19	-0.0012	0.0006	0.0093	0.0033	0.0102	0.0041	-8.38	29	.549	0.72	-0.0022	0.0005
32.50	0.0070	0.0026	0.0075	0.0024	-5.82	-0.93	.634	-0.25	-0.0014	0.0006	0.0092	0.0033	0.0099	0.0038	-7.16	40	.789	0.62	-0.0019	0.0004
32.75	0.0068	0.0026	0.0073	0.0023	-6.38	-1.13	.593	-0.30	-0.0014	0.0004	0.0091	0.0035	0.0098	0.0035	-6.93	37	.732	0.65	-0.0019	0.0006
33.00	0.0067	0.0026	0.0070	0.0022	-3.30	-0.65	.705	-0.17	-0.0010	0.0005	0.0090	0.0034	0.0098	0.0033	-7.61	32	.606	0.70	-0.0020	0.0005
33.25	0.0067	0.0026	0.0067	0.0021	-0.85	-0.17	.929	-0.04	-0.0008	0.0007	0.0088	0.0033	0.0097	0.0033	-9.11	25	.352	0.76	-0.0020	0.0002

33.50	0.0067	0.0026	0.0068	0.0022	-2.50	-0.43	.814	-0.11	-0.0010	0.0007	0.0087	0.0033	0.0097	0.0033	-10.48	24	.343	0.77	-0.0022	0.0002
33.75	0.0065	0.0024	0.0069	0.0022	-4.84	-0.75	.656	-0.20	-0.0013	0.0006	0.0086	0.0031	0.0096	0.0032	-10.67	30	.549	0.71	-0.0023	0.0002
34.00	0.0064	0.0024	0.0069	0.0022	-7.35	-1.16	.593	-0.31	-0.0015	0.0004	0.0085	0.0029	0.0095	0.0030	-11.38	-1.94	.343	-0.52	-0.0023	0.0001
34.25	0.0064	0.0024	0.0070	0.0024	-8.72	38	.634	0.64	-0.0016	0.0004	0.0083	0.0028	0.0093	0.0030	-10.96	24	.343	0.77	-0.0020	0.0000
34.50	0.0062	0.0023	0.0065	0.0021	-4.47	-0.73	.667	-0.19	-0.0012	0.0006	0.0082	0.0028	0.0091	0.0032	-10.54	24	.343	0.77	-0.0020	0.0001
34.75	0.0060	0.0022	0.0060	0.0018	0.01	0.00	1.000	0.00	-0.0006	0.0007	0.0080	0.0027	0.0090	0.0032	-10.66	18	.172	0.83	-0.0020	0.0001
35.00	0.0058	0.0021	0.0059	0.0017	-1.26	-0.28	.908	-0.07	-0.0006	0.0005	0.0080	0.0026	0.0088	0.0032	-8.90	25	.352	0.76	-0.0018	0.0002

PSG = polysomnography. Diff = percentage difference between STIM and SHAM. Positive difference scores indicate higher spectral power in STIM compared to SHAM.
